# Sirtuins and tumor immunity: mechanistic insights, immunotherapy prospects, and therapeutic horizons

**DOI:** 10.3389/fimmu.2025.1700483

**Published:** 2025-12-04

**Authors:** Jinning Gu, Shanshan Liu, Wen Xiao, Wei Qu

**Affiliations:** 1 Department of General Medicine, The Second Hospital of Jilin University, Changchun, China; 2 Department of Ultrasound, The Second Hospital of Jilin University, Changchun, China

**Keywords:** sirtuins, oncogenic processes, immunotherapy, immune escape, chemoresistance

## Abstract

Sirtuins (SIRTs), a family of NAD^+^-dependent enzymes, exhibit complex and sometimes opposing functions in cancer biology. These enzymes can function as tumor suppressors or promoters, depending on the cellular context, tumor type, and metabolic state. This review provides a mechanistic overview of SIRT isoform regulation of key oncogenic processes, including proliferation, metastasis, metabolic reprogramming, and chemotherapy resistance. Special emphasis is given to their immunomodulatory roles within the tumor microenvironment (TME), where SIRTs influence T cell differentiation, immune checkpoint expression, macrophage polarization, and natural killer cell function. SIRT-driven pathways, such as the nicotinamide phosphoribosyltransferase (NAMPT)–SIRT1–programmed Cell Death Ligand 1 (PD-L1) axis, SIRT6-induced regulatory T cell (Treg) formation, and SIRT2-driven T cell activation, are examined for their effects on immune escape or enhancement and their impact on immunotherapy responses. The review also explores how SIRTs contribute to adaptive mechanisms underlying chemoresistance, including autophagy, epithelial-mesenchymal transition (EMT), redox balance, and mitochondrial protection. The therapeutic landscape of targeting SIRTs is assessed, with discussion of isoform-selective modulators, combination strategies with checkpoint blockade, and challenges in leveraging their context-dependent activities. SIRTs are established as crucial regulators of cancer immunity and therapy, suggesting novel directions for precision oncology. However, given their isoform- and context-dependent duality across tumor types, the clinical translation of SIRT modulators requires careful mechanistic stratification and biomarker-guided patient selection.

## 1 Introduction

Cancer is a multifactorial disease characterized by the convergence of genetic mutations (1, 2), epigenetic modifications (3, 4), metabolic reprogramming (5, 6), and immune dysfunction within a dynamic tumor microenvironment (TME) (1, 2). Among the plethora of molecular modulators that orchestrate these events, SIRTs are an evolutionarily conserved family of NAD^+^-dependent deacetylases and ADP-ribosyltransferases that have emerged as critical, yet enigmatic, regulators of tumorigenesis (3–6). These enzymes control a wide range of cellular processes, ranging from chromatin structure remodeling (7, 8) and DNA repair (9, 10) to mitochondrial homeostasis (11, 12) and immune cell differentiation (13, 14), and hence constitute the primary sensors and regulators of cellular stress, metabolism, and longevity.

Over recent years, the role of SIRTs in cancer has undergone significant shifts. Initially defined by their tumor-suppressing functions, which maintain genome stability (15), limit metabolism (16), and trigger apoptosis (15), SIRTs are now recognized for their context-dependent influence, which can either inhibit or promote tumorigenesis. This duality is primarily governed by isoform specificity, subcellular distribution, and the cancer’s biochemical environment. Isoforms such as SIRT1, SIRT2, and SIRT6 are mainly associated with tumor-suppressive activities, a function determined by their subcellular localization and the biochemical context of each cancer type (3, 16–18).

Along with the intrinsic control of tumor cells, SIRTs orchestrate extrinsic signals that determine the immunological context of the TME. SIRTs modulate T cell differentiation (19, 20), influence the activity of natural killer (NK) cells (21), reprogram macrophage states (22), and modulate immune checkpoint expression (23); in this manner, they directly influence the effectiveness of immunotherapies (24, 25). Concurrently, SIRTs regulate adaptive processes, such as autophagy, epithelial–mesenchymal transition (EMT), and redox homeostasis, allowing tumor cells to resist chemotherapy-induced cytotoxicity (26–28). Such findings place SIRTs not only as markers for cancer progression and responsiveness to therapy, but also as candidate therapeutic targets. This review places recent mechanistic findings into the multifunctional role of SIRTs in cancer, their regulation of cell proliferation, metabolic adaptation, autophagy, immunity, responsiveness to immunotherapy, and chemoresistance. By integrating evidence from various cancers and pathways, we delineate herein the dualistic function of SIRTs and discuss their promise as modulators of cancer therapy in the era of precision oncology.

## 2 Overview of sirtuin structure, function, and their roles in health and disease

SIRTs are a family of evolutionarily conserved NAD+-dependent enzymes represented in several species. SIRTs regulate chromatin remodeling, transcriptional control, and signaling cascades across prokaryotic and eukaryotic systems (29). All seven isoforms share a conserved deacylation mechanism comprising four sequential steps: substrate binding, glycosidic bond cleavage, acetyl transfer, and product formation (29–31). Structural analysis of SIRTs through X-ray crystallography has revealed that the catalytic core comprises two bilobed globular domains, each containing approximately 275 amino acid residues and requiring the cofactor NAD for enzymatic activity. The N- and C-terminal parts of SIRT proteins exhibit significant differences in terms of size, chemical composition, sensitivity to post-translational modifications such as phosphorylation, and their substrate-binding ability (32–34).

**Figure 1:**
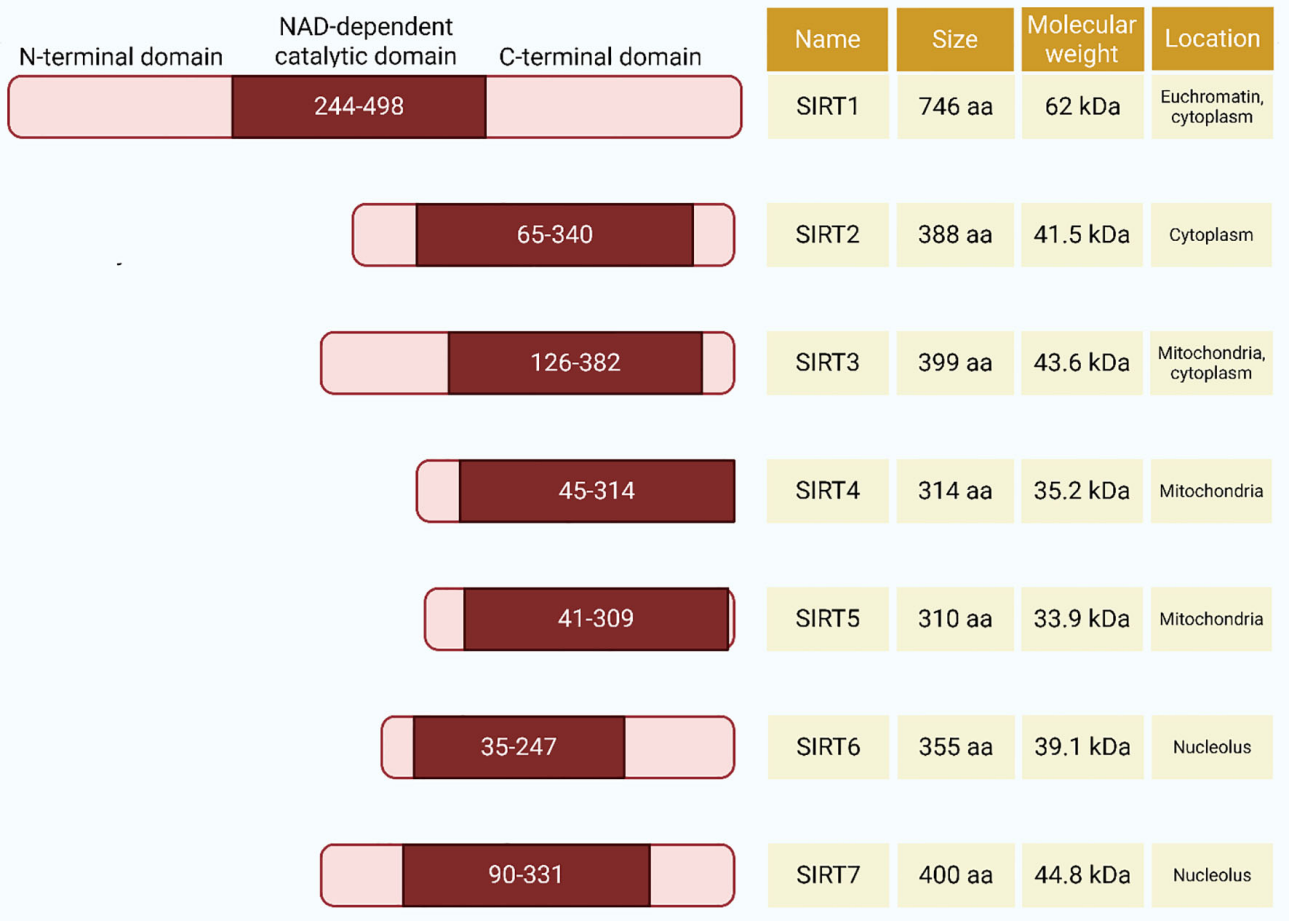
Domain organization, subcellular localization, and molecular weight of human sirtuins (SIRT1–SIRT7). Each sirtuin protein is represented with its N-terminal domain, NAD^+^-dependent catalytic domain, and C-terminal domain. The amino acid positions flanking each domain are indicated. Subcellular localization and molecular weight (in kDa) of each isoform are shown on the right. Notably, SIRT1 is the largest isoform, located in euchromatin and cytoplasm, while SIRT4 and SIRT5 are exclusively mitochondrial, and SIRT6 and SIRT7 localize to the nucleolus.

In contrast to this structural heterogeneity, the catalytic core sequences of SIRTs are exceedingly conserved, exhibiting extremely high structural homology. Such a core domain consists of: (a) Rossmann-fold NAD-binding domain of large size; (b) variable small zinc-binding domains; and (c) flexible loop domains between the Rossmann-fold domain and the zinc-binding domains (35, 36). The loops create distinct extended clefts between the large and small domains, serving as entry points for NAD and acetyl-lysine-containing peptide substrates, which bind to the enzyme from opposite directions. The amino acid residues responsible for catalysis and the binding of the two substrate molecules are located within protein tunnels formed in the inter-domain spaces (36). Of the seven SIRT isoforms, their homologous catalytic cores employ a conserved deacylation mechanism, which is an underlying similarity. The mechanism follows the following sequential steps: (a) NAD and acetylated lysine substrate binding; (b) glycosidic bond cleavage; (c) acetyl group transfer; and (d) reaction product formation of O-acetyl-ADP-ribose, nicotinamide, and deacetylated lysine residue (**Figure 1**) (29).

These differences in the members of the SIRT protein family were first attributed to their respective subcellular locations (37). Specifically, SIRT1 predominantly resides in the nucleus but can translocate to the cytoplasm under certain conditions (38, 39). SIRT2 is localized mainly in the cytosol but also localizes in the nucleus during the transition of the cell cycle from G2 to M phase (40). Members of the SIRT3 to SIRT5 family are mainly mitochondria-localized, as they contain mitochondrial localization signals (41–43). In contrast, SIRT6 and SIRT7 are nuclear proteins: SIRT6 is largely chromatin-bound, while SIRT7 is located largely in the nucleolus (17, 44). In addition to their cell location, another important difference between members of the SIRT family is in their enzymatic capabilities. Mammalian SIRTs have an expanded functional repertoire, with SIRT1, SIRT2, and SIRT3 exhibiting robust deacetylase activity. SIRT4 is primarily an ADP-ribosyltransferase that controls β-cell glutamate dehydrogenase activity, thereby limiting insulin secretion (42). SIRT5 is a lysine malonylation, succinylation, and glutarylation post-translational modification enzyme (45, 46). SIRT6 is also an NAD+-dependent mono-ADP-ribosyltransferase and a long-chain fatty acyl deacetylase enzyme (47, 48). SIRT7 is a deacetylase and is primarily localized in the nucleolus, where it plays a key role in modulating RNA polymerase I transcription (49). It has been found that SIRTs can target a vast range of proteins, including both histone and non-histone substrates. By means of such modifications, they are implicated in the regulation of numerous important cellular processes, including glucose and lipid metabolism, mitochondrial biogenesis, DNA repair pathways, response to oxidative stress, apoptosis, and inflammation (50). In short, the pleiotropic enzymatic activities of SIRTs enable them to alter key biological pathways by acting on proteins that play crucial roles in modulating metabolism, genomic integrity, redox balance, and inflammatory signaling.

The physiological functions of the SIRTs overlap with those of several systems. SIRTs are involved in silencing genes (34, 51), regulation of the cell cycle (52), cellular metabolism (53–55), and the modulation of apoptosis (56, 57), longevity (58–60), and neurobehavioral functions such as mood, cognition, and aging (61–63). Under normal physiological conditions, SIRTs are involved in healthy aging (64), resistance to stress (65), regulation of circadian rhythms (66), as well as immune homeostasis (67). Nonetheless, sirtuin dysregulation of expression or activity underlies a vast majority of pathological conditions, including metabolic syndrome (68, 69), neurodegenerative diseases (70, 71), cardiovascular diseases (68, 69), and cancer (70, 71). The nuclear receptor NR3C2 induces SIRT1 expression in colorectal cancer (CRC), forming an effective NR3C2–SIRT1 axis (70). They demonstrated that NR3C2 upregulates SIRT1, which subsequently elevates LC3B and p62 expression. This pathway significantly repressed CRC cell invasion and lung metastasis (70). These observations confirm the tumor-suppressive effect of SIRT1, which is mediated by inducing autophagy.

Over the past decade, SIRTs have come into the spotlight as indicators of immune and metabolic function (72, 73) and have even been identified as therapeutic targets. Pharmacologic modulators—activators and inhibitors are being explored in the preclinical and clinical settings for their ability to modulate SIRT under disease-specific contexts (74). A comprehensive understanding of their context-dependent functions is crucial for enabling the rational design of targeted interventions that leverage the dual role of SIRTs in cell homeostasis and disease.

## 3 Mechanistic roles of sirtuins in cancer: proliferation, autophagy, and glycolysis

SIRTs have become context-dependent modulators of tumor cell biology. Their mechanistic function transcends classical pathways and increasingly overlaps with pivotal processes that characterize malignancy. These include cancer cell proliferation and invasion, autophagy, and metabolic reprogramming through glycolysis, which serve as central axes of SIRT activity. The combined contributions of these processes enable tumor growth and resistance to therapy. Recent research has explained the dualistic functions of SIRT in these contexts. According to isoform, tissue microenvironment, and upstream molecules, SIRTs tend to enhance or inhibit tumorigenic programs. In proliferation and metastasis, SIRTs cross-talk with transcriptional and post-translational pathways to modulate EMT, angiogenesis, and chromosomal stability. Autophagy, the second life-or-death mechanism regulated by SIRTs, appears either as a survival or death signal, frequently deciding treatments. Furthermore, SIRTs are key regulators of glycolytic reprogramming, a characteristic of cancer metabolism, either stimulating glucose flow to facilitate proliferation or inhibiting it to maintain redox balance and prevent drug resistance. In the following sections, we integrate recent evidence that chronicles the diverse roles of SIRTs in these three areas and highlight their integration into cancer cell metabolism, growth, and survival pathways. Together, these results locate SIRTs at nodes of significance for therapy in numerous cancers.

### 3.1 Proliferation, invasion, and metastasis

**Figure 2:**
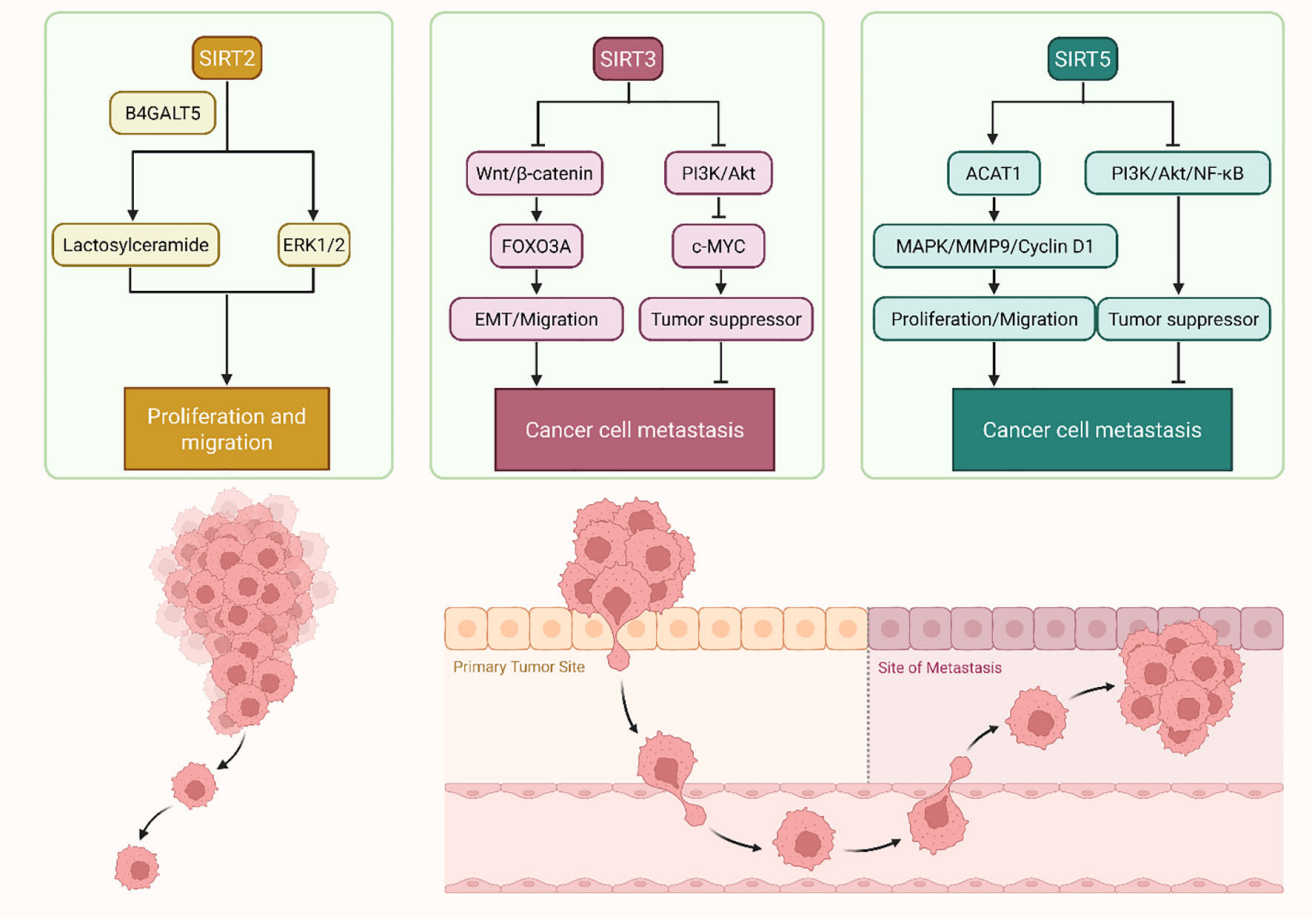
Divergent roles of SIRT2, SIRT3, and SIRT5 in regulating cancer cell metastasis through distinct molecular mechanisms. SIRT2 promotes cancer cell proliferation and migration via B4GALT5-mediated lactosylceramide synthesis and ERK1/2 signaling. SIRT3 exerts tumor-suppressive effects by activating FOXO3A and suppressing c-MYC through Wnt/β-catenin and PI3K/Akt pathways, thereby inhibiting EMT and migration. In contrast, SIRT5 displays dual roles: it enhances proliferation and migration through ACAT1–MAPK–MMP9/cyclin D1 signaling, while simultaneously suppressing metastasis via PI3K/AKT/NF-κB inhibition. The lower panel illustrates the corresponding phenotypic consequences on cancer cell metastatic behavior. In NSCLC, SIRT1 exhibits a dual program: HNRNPD-biased SIRT1/PGC-1α signaling promotes vasculogenic mimicry and invasion, whereas SIRT1-mediated FOXO1 activation suppresses proliferation and induces apoptosis. Metabolic (NAMPT/PARP) and AMPK-dependent energy cues modulate this switch.

SIRTs have various and context-dependent impacts on tumor cell behavior. Sirtuins have dualistic functions in proliferation, invasion, and metastasis of various cancers (**Figure 2**, **Table 1**). SIRTs of non-small cell lung carcinoma (NSCLC) exhibit both tumor-suppressive and tumor-promoting functions, depending on their molecular interactions. In NSCLC, distinct upstream regulators define the dual role of SIRT1. Activation of the Heterogeneous Nuclear Ribonucleoprotein D (hnRNP D)/PPARG Coactivator 1 Alpha (PGC-1α) module enhances angiogenesis and invasion, whereas FOXO1-driven acetylation following SIRT1 loss suppresses tumor growth and promotes apoptosis (88, 90). Pharmacological inhibition of FOXO1 would reverse this, suggesting that SIRT1 inhibits NSCLC malignancy via the FOXO pathway. This duality suggests a possible molecular switch in SIRT1 activity in NSCLC (88). The functional switch between these programs—identified in NSCLC through HNRNPD/PGC-1α (83) and FOXO1 (97) —appears to be further modulated by general SIRT1 regulatory layers operating across various cancer contexts. These include: (i) transcriptional co-regulator dominance, where the relative availability of HNRNPD/PGC-1α versus FOXO1 determines the downstream transcriptional outcome; (ii) metabolic regulation of NAD^+^ flux, governed by the NAMPT–poly(ADP-ribose) polymerase (PARP) axis, which controls SIRT1’s catalytic capacity (24), energy-stress signaling via AMP-activated protein kinase (AMPK), which couples mitochondrial apoptosis and therapeutic sensitivity to SIRT1 activity (113). These parameters define a metabolic–transcriptional rheostat that determines whether SIRT1 promotes oncogenic or tumor-suppressive outcomes in NSCLC. The apparently contradictory effects of SIRT1 in NSCLC reflect context-specific routing through HNRNPD/PGC-1α-driven pro-angiogenic versus FOXO1-dependent tumor-suppressive networks. Additional complexity arises with SIRT6, which deacetylates and stabilizes the anti-metastatic protein GILZ, preventing its ubiquitin-mediated degradation (92). This stabilization inhibits EMT, consequently inhibiting cell migration and invasion (92). Collectively, these results position SIRT1 and SIRT6 at opposite poles of the NSCLC metastatic spectrum, with their regulatory functions firmly linked to interacting protein networks.

**Table 1:** Molecular mechanisms and oncological outcomes of SIRT modulation across diverse cancer types.

Cancer type	Study type	Mechanism	Conclusion	Ref.
CRC	*In vitro* and *in vivo*	circ-SIRT1 binds EIF4A3 to regulate EMT markers	circ-SIRT1 promotes CRC proliferation and EMT via EIF4A3 pathway	(75)
CRC	*In vivo* and *in vitro*	miR-212-5p downregulates SIRT2 to promote proliferation/metastasis	SIRT2 acts as tumor suppressor, downregulated by miR-212-5p	(76)
CRC	*In vitro* and *in vivo*	SIRT2 deacetylates IDH1, affecting α-KG and HIF1α-SRC pathway	SIRT2 suppresses CRC metastasis via IDH1 deacetylation	(77)
CRC	*In vitro* and patients sample	SIRT1 suppresses CRC metastasis by blocking miR-15b-5p transcription	SIRT1 inhibits CRC metastasis through SIRT1/miR-15b-5p/ACOX1 axis.	(78)
Papillary thyroid carcinoma	*In vitro* and *in vivo*	SIRT4 overexpression suppresses EMT via E-cadherin/N-cadherin modulation	SIRT4 suppresses PTC growth and invasion	(79)
Breast cancer	*In vitro*	SIRT4 promotes proliferation/migration/invasion	SIRT4 shows oncogenic role in breast cancer	(80)
Breast cancer	*In vitro* and *in vivo*	SIRT6 supports DNA repair; β-carboline derivative inhibits SIRT6 to induce apoptosis	SIRT6 inhibition induces breast cancer apoptosis via DNA repair block	(81)
Breast cancer	*In vitro* and *in vivo*	SIRT7 knockdown induces CIN via LAP2α degradation	SIRT7 deficiency leads to metastasis via CIN in breast cancer	(82)
Breast cancer	*In vitro*	SIRT2 inhibition → ↓invasion in TNBC	AGK2 reduces TNBC migration by inhibiting SIRT2	(83)
Glioma	*In vitro* and *in vivo*	BLA targets SIRT6, inhibits histone acetylation and glioma cell proliferation	BLA inhibits glioma by targeting SIRT6	(84)
Glioma	*In vitro* and *in vivo*	SIRT5 regulates mitochondrial metabolism and synaptic remodeling	SIRT5 suppresses glioma growth via metabolism and plasticity	(85)
Glioma	*In vitro* and *in vivo*	SIRT1 deacetylates FOXO1 → ↑androgen synthesis	SIRT1 deacetylates FOXO1 to regulate GBM androgens	(86)
Glioma	*In vitro*	SIRT7 → ERK/STAT3 → ↑proliferation/invasion	SIRT7 promotes glioma via ERK/STAT3 activation	(87)
NSCLC	*In vitro* and *in vivo*	SIRT1 silencing activates FOXO1 to inhibit malignancy	SIRT1 inhibition suppresses NSCLC via FOXO1 activation	(88)
NSCLC	*In vitro*	SIRT3 overexpression increases ROS to inhibit NSCLC cell growth	SIRT3 acts as tumor suppressor via ROS induction	(89)
NSCLC	*In vitro* and *in vivo*	HNRNPD activates SIRT1/PGC-1α promoting vasculogenic mimicry	SIRT1/PGC-1α pathway promotes NSCLC vasculogenic mimicry	(90)
NSCLC	*In vivo* and *in vitro*	Betaxanthin inhibits insulin-driven proliferation via SIRT3 upregulation	SIRT3 mediates BET anti-tumor effect in obesity-linked lung cancer	(91)
NSCLC	*In vitro*	SIRT6 deacetylates and stabilizes GILZ to suppress EMT	SIRT6 stabilizes GILZ to suppress NSCLC cell migration	(92)
NSCLC	*In vitro* and *in vivo*	SIRT6 inhibits PI3K/Akt/mTOR signaling to enhance radiosensitivity	SIRT6 promotes radiosensitivity via PI3K/Akt/mTOR inhibition	(93)
NSCLC	*In vitro*	SIRT6 knockdown → ↑acetyl-DNMT1 → NOTCH1 repression	SIRT6 silencing represses NOTCH signaling, inhibits proliferation	(94)
Lung cancer	*In vitro*	SIRT5 de-succinylates HIST1H2BL; lidocaine reduces its level	Lidocaine suppresses NSCLC via SIRT5-HIST1H2BL axis	(95)
ESCC	*In vitro* and *in vivo*	SIRT2 deacetylates ACLY enhancing ESCC progression	SIRT2/ACLY axis promotes ESCC progression	(96)
PCa	*In vitro*	SIRT2 activates ERK1/2 and lactosylceramide production via B4GALT5	SIRT2 promotes proliferation and migration in prostate cancer	(97)
PCa	*In vitro* and *in vivo*	SIRT3 inhibits Wnt/β-catenin → ↑FOXO3A → ↓EMT/migration	SIRT3 suppresses EMT and metastasis via Wnt/β-catenin-FOXO3A	(98)
PCa	*In vitro* and *in vivo*	SIRT3 suppresses PI3K/Akt → ↓c-MYC	SIRT3 acts as tumor suppressor via PI3K/Akt inhibition	(99)
PCa	*In vitro*	SIRT5 → ACAT1 → ↑MAPK → ↑MMP9 and cyclin D1	SIRT5 promotes proliferation/migration via MAPK signaling	(100)
PCa	*In vitro*	SIRT5 inhibits PI3K/AKT/NF-κB	SIRT5 suppresses metastasis via PI3K/AKT/NF-κB inhibition	(101)
PCa	*In vitro*	SIRT6 activates Wnt/β-catenin	SIRT6 enhances proliferation via Wnt/β-catenin pathway	(102)
HCC	*In vitro*	HBx-induced ↑SIRT1 → ↑proliferation/migration	HBx-induced SIRT1 promotes HCC tumorigenesis	(103)
HCC	*In vitro* and *in vivo*	GNL3 regulates SIRT1, induces stemness and metastasis	GNL3 via SIRT1 induces metastasis and stemness	(104)
HCC	*In vitro*	SIRT5 activates E2F1 → ↑proliferation/invasion	SIRT5 promotes HCC progression via E2F1	(105)
HCC	*In vitro* and *in vivo*	SIRT7 suppresses MST1 → ↑YAP activity	SIRT7 suppresses MST1 → activates YAP in HCC	(106)
Melanoma	*In vitro* and *in vivo*	USP22 → SIRT1/PTEN/PI3K → ↑metastasis	USP22 via SIRT1/PTEN enhances melanoma metastasis	(107)
Melanoma	*In vivo*	SIRT1/SIRT3 dual inhibition → ↓melanoma progression	Dual SIRT1/3 inhibition suppresses melanoma	(108)
Melanoma	*In vivo*	SIRT5 → histone acylation → ↑MITF/c-MYC	SIRT5 maintains chromatin and oncogene expression	(109)
Melanoma and Lung cancer	*In vitro* and *in vivo*	Arg-II suppresses SIRT3 → ↑mtROS → ↑growth, migration, nuclear deformation, DNA damage	Arg-II/SIRT3/mtROS axis promotes melanoma and lung cancer progression and malignancy	(110)
Gastric cancer	*In vitro*	RACGAP1 inhibits SIRT1/Mfn2 → ↓autophagy	RACGAP1 represses SIRT1, inhibits autophagy	(111)
Gastric cancer	*In vitro*	SIRT2 → PEPCK1 → RAS/ERK/JNK/MMP-9 pathway	SIRT2 enhances GC migration/invasion via metabolism	(112)

In brain tumors, SIRTs are largely tumor-suppressive as well. Experimental studies in glioma models revealed that SIRT6 overexpression suppresses tumor growth by promoting histone deacetylation and cell-cycle arrest, whereas SIRT1 limits androgen biosynthesis through FOXO1 deacetylation, thereby restraining tumor progression (84, 86). SIRT1 overexpression decreased intracranial androgen and suppressed GBM growth, implicating a tumor-suppressive activity by endocrine regulation (86).

In CRC, SIRT1 mediates β-catenin deacetylation, which drives its nuclear exclusion and attenuates the Wnt signaling pathway (114). This places SIRT1 as a downstream effector of vitamin D/VDR signaling, thus connecting dietary and genetic modulation of tumor behavior. Conversely, SIRT2 is a tumor suppressor in CRC and is repressed by miR-212-5p (76). Its overexpression repressed cell growth and metastasis, again suggesting its therapeutic potential (76). In ESCC, however, Zhang et al. have found that SIRT2 promotes tumor formation by deacetylating ACLY, thus advancing lipid metabolism, cell migration, and invasion (96). Therefore, SIRT2 fulfills opposite roles in CRC and ESCC, illustrating context dependency even within the digestive tract (96). Yan et al. have observed that in gastric cancer, RACGAP1 suppresses SIRT1/Mfn2, thus advancing cell proliferation, invasion, and survival (111). Reconstitution of SIRT1 expression triggered apoptosis and autophagy, further confirming its anti-cancer action in this case (111).

The roles of SIRTs in hormone-related cancers are likewise dualistic. Liang et al. demonstrated that SIRT6 is an oncogene in breast cancer, promoting DNA repair and chemoresistance (81). SIRT6 selective inhibition with a β-carboline scaffold induced enhanced apoptosis and decreased invasion (81). Alternatively, Huo et al. showed that downregulation of SIRT7 in breast cancer destabilizes LAP2α and generates CIN and metastasis (82). Reinforcement of SIRT7 suppressed these processes, suggesting that, unlike SIRT6, SIRT7 functions to maintain genomic integrity and prevent spreading (82). Lastly, Jessop et al. investigated the function of SIRT2 in TNBC (83). Pharmacological inhibition of SIRT2 remodeled the perinuclear cytoskeleton and hardened nuclear architecture, diminishing invasiveness in small spaces (83). These results suggest a specific biomechanical function of SIRT2 in promoting metastasis. In prostate cancer (PCa), Lin et al. found that SIRT2 promotes proliferation and invasion by inducing extracellular signal-regulated kinase (ERK)1/2 activation and biosynthesis of lactosylceramide by B4GALT5 (97). These signal transduction and metabolic alterations were associated with castration-resistant neuroendocrine differentiation (97).

In melanoma, Yang et al. described a pathway by which arginase-II suppressed the expression of SIRT3, leading to elevated amounts of mitochondrial ROS, DNA damage, and increased metastatic potential (110). Reconstitution of SIRT3 blocked these activities, suggesting mitochondrial regulation as an important metastatic control pathway (110). In parallel, Sun et al. demonstrated that USP22 promotes melanoma metastasis by activating the SIRT1/PTEN/PI3K pathway (107). Pharmacological USP22 inhibition decreased lung metastases and increased ferroptosis sensitivity, demonstrating the tractability of SIRT-related pathways in oncology medicine (107). Gu et al. reported that SIRT7 inhibits MST1 transcriptionally and post-transcriptionally, enabling YAP activation and oncogenic transcription in liver cancer (106). Inhibition of SIRT7 reversed MST1 levels and inhibited hepatocellular carcinoma (HCC) growth, demonstrating the SIRT7–MST1–YAP axis as a target (106).

Together, these studies demonstrate that the functional topography of SIRTs in cancer is not solely defined by isoform identity but also by their integration into specific cellular and molecular contexts. While SIRT1, SIRT2, and SIRT6 can assume opposite functions in relation to the tumor context, SIRT3, SIRT4, and SIRT7 tend to function more in favor of tumors. These functions are controlled through the modulation of numerous processes, including EMT, mitochondrial ROS homeostasis, lipid metabolism, and maintenance of genomic integrity. Many other new original research findings, which validate and expand on these mechanistic findings, are listed in **Table 1**. Cumulatively, this body of evidence supports the significance of context-dependent analysis of SIRT biology towards the aim of developing targeted therapy for cancer.

### 3.2 Cell autophagy

**Figure 3:**
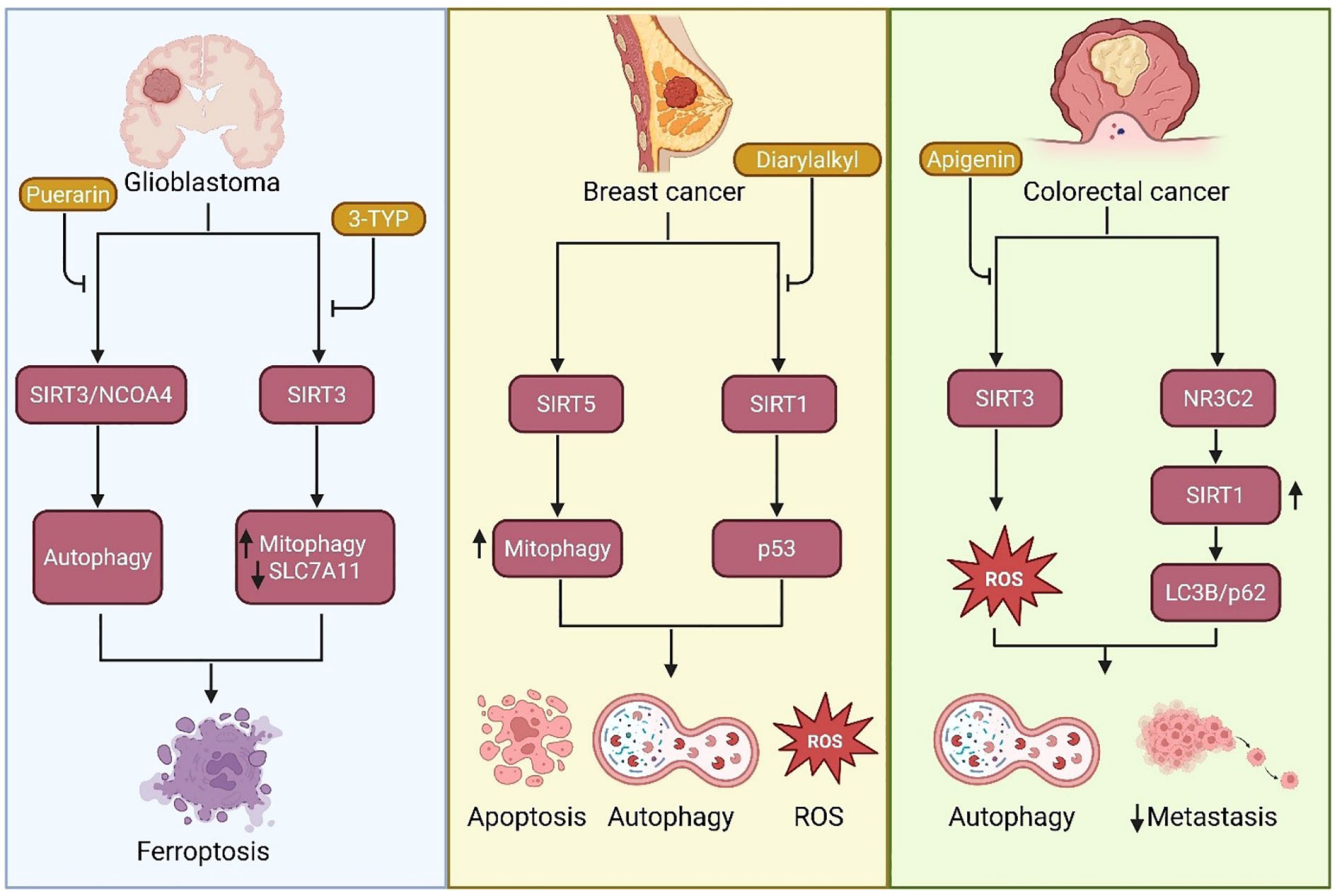
SIRT-regulated autophagy and ferroptosis pathways in glioblastoma, breast cancer, and colorectal cancer (CRC) cells. In glioblastoma, Puerarin activates the SIRT3/NCOA4 axis to promote ferroptosis through autophagy, while 3-TYP inhibits SIRT3, enhancing mitophagy and downregulating SLC7A11 to increase ferroptosis. In breast cancer, Diarylalkyl compounds modulate SIRT1 and SIRT5 activity; SIRT1 induces autophagy and apoptosis via p53, whereas SIRT5 suppression enhances ROS production and autophagy through reduced mitophagy. In CRC cells, Apigenin induces ROS-dependent autophagy via SIRT3 activation, and NR3C2-driven upregulation of SIRT1 promotes LC3B/p62-mediated autophagy, inhibiting EMT and metastasis. Bottom panels illustrate the associated cellular outcomes.

SIRTs are newly recognized key modulators of cancer cell fate by regulating autophagy (**Figure 3**, **Table 2**). Recent research has revealed context-dependent roles for specific SIRTs, associating them with tumor suppression or growth through unique signaling pathways. In CRC, activation of the NR3C2–SIRT1 axis promotes autophagy through LC3B lipidation and p62 upregulation, while concurrently suppressing EMT (70). Mechanistically, SIRT1 interacts with the promoters of autophagy-associated genes, promoting chromatin remodeling and thereby initiating a transcriptional program that promotes autophagic flux and inhibits metastatic capability (70). In contrast, Abdelmaksoud et al. found that the flavonoid apigenin downregulates mitochondrial SIRT3 in a mouse CRC model, resulting in decreased MnSOD and increased ROS generation (115). Elevated ROS triggers JNK signaling, which promotes HMGB1 translocation and Beclin 1 overexpression, resulting in autophagy (115). This demonstrates a redox-dependent, SIRT3-mediated regulatory circuit in determining cancer cell fate.

**Table 2:** Sirtuin-mediated autophagy pathways and their tumor-specific functional consequences across cancer types.

Cancer type	Study type	Autophagy mechanism	Study conclusion	Ref.
CRC	*In vitro* and *in vivo*	NR3C2–SIRT1 axis upregulates LC3B/p62	Promotes autophagy and inhibits EMT/metastasis	(70)
CRC	*In vivo* (mouse model)	SIRT3 inhibition → ROS → HMGB1/Beclin1	Apigenin promotes ROS-dependent autophagy	(115)
CRC	*In vitro* and *in vivo*	SIRT3/Hsp90/AKT pathway	MY-13 induces autophagy-dependent cell death	(116)
Lung cancer	*In vitro*	SIRT1/AMPK signaling	Quercetin induces autophagic apoptosis	(117)
Lung cancer	*In vitro*	SIRT1/2 inhibition → ATF4-DDIT4-mTORC1	Pro-survival autophagy via ER stress	(118)
Lung cancer	*In vitro*	SIRT3 → ↓PI3K/AKT/mTOR	Autophagy protects against DOX-induced senescence	(119)
HCC	*In vitro* and patient data	SIRT2-mediated ANXA2 deacetylation → ↓mTOR	Protective autophagy, drug resistance	(120)
Liver cancer	*In vitro*	SIRT5 deacetylates cytochrome c	Promotes mitochondrial apoptosis	(121)
Liver cancer	*In vitro* and *in vivo*	SIRT1-regulated autophagy and inflammation	Oncogenic effects in hepatocarcinogenesis	(122)
Oral squamous cell carcinoma	*In vitro*	SIRT3/AMPK/mTOR/ATG16L1	Autophagy promotes ferroptosis	(123)
Pancreatic cancer	*In vitro* and *in vivo*	SIRT4 → AMPKα → p53	Autophagy inhibits tumorigenesis	(124)
Breast cancer	*In vitro*	SIRT5 activation → ↓mitophagy	ROS accumulation, autophagy modulation	(125)
Breast cancer	*In vitro*	SIRT1 inhibition → p53 activation	Induces autophagy and apoptosis	(126)
Gastric cancer	*In vitro*	miR-543 targets SIRT1	Inhibits autophagy, promotes EMT	(127)
Cervical cancer	*In vitro* and *in vivo*	SIRT7 → USP39/FOXM1 axis	Autophagy activation and ROS suppression	(128)
PCa	*In vitro*	Autophagy-mediated SIRT1 downregulation	Increases radiosensitivity	(129)
PCa	*In vitro* and *in vivo*	SIRT7 → AR/SMAD4	Androgen-induced autophagy	(130)
Various cancer types	*In vitro*	SIRT6 → ROS → AMPK–ULK1–mTOR	Triggers lethal autophagy	(131)
Leukemia	*In vitro* and *in vivo*	SIRT1 knockdown → HMGB1/ACSL4	Reverses drug resistance via ferroptosis	(132)
GBM	*In vitro* and *in vivo*	SIRT5 → BCAT1 desuccinylation	Inhibits ferroptosis, promotes proliferation	(133)
GBM	*In vitro* and *in vivo*	SIRT3 inhibition → ↑mitophagy, ↓SLC7A11	Enhances ferroptosis	(134)
GBM	*In vitro*	Puerarin → SIRT3/NCOA4	Promotes ferroptosis through autophagy	(135)

Mou et al. presented MY-13, a novel SIRT3 activator that inhibits CRC cell growth through apoptosis and autophagy (116). MY-13 increases SIRT3 activity, leading to the deacetylation and inactivation of Hsp90, the instability of AKT, and the subsequent activation of autophagic death pathways (116). This study delineates a SIRT3/Hsp90/AKT axis that governs cell survival (116). Similarly, in oral squamous cell carcinoma, SIRT3 promotes ferroptotic cell death through the activation of AMPK/mTOR signaling and ATG16L1-mediated autophagy, especially under quercetin treatment (123). Sun et al. unveiled SIRT2 as mediating the deacetylation of ANXA2 in HCC, thereby inhibiting mTOR activity and eliciting protective autophagy, which is responsible for donafenib resistance, a perfect example of autophagy as a survival strategy (120). At the same time, Fan et al. demonstrated that SIRT3 rescues doxorubicin-induced senescence in A549 lung cancer cells by restoring autophagic flux and inhibiting the PI3K/AKT/mTOR pathway, implicating SIRT3 in the regulation of oxidative stress and anti-aging functions (119).

Li et al. studied SIRT4 in pancreatic ductal adenocarcinoma and demonstrated that SIRT4-induced autophagy occurs through the repression of glutamine metabolism, leading to enhanced p53 phosphorylation via AMPKα activation (124). This autophagy pathway presents decreased tumorigenesis, validating a metabolic rationale for SIRT4-targeted therapy (124). Barreca et al. studied SIRT5 activation in breast cancer employing a synthetic SIRT5 activator, MC3138. SIRT5-mediated deacetylation suppresses glutaminase (GLS) activity, decreasing ammonia generation and mitophagy (125). Increased ROS caused by defective mitochondrial clearance leads to cytotoxicity, demonstrating the anti-tumor activity of SIRT5-autophagy and redox regulation (125). Derivatives of 2-(diarylalkyl)aminobenzothiazole suppress SIRT1 in breast cancer cells, thus enhancing p53 acetylation. This activates autophagy and apoptosis in MCF7 cells, indicating that SIRT1 inhibition unleashes pro-death signals by exposing p53 activity and LC3-II aggregation (126). Yu et al. demonstrated that SIRT7 participates in a positive feedback cycle with USP39 and FOXM1 in cervical squamous cell carcinoma, promoting autophagy and lowering ROS levels. SIRT7 deacetylates and stabilizes USP39, enhancing FOXM1 transcriptional activity and thereby stimulating tumor growth through the autophagic regulation of redox homeostasis (128).

Iachettini et al. demonstrated that SIRT6 pharmacological activation by UBCS039 triggers an ROS burst, resulting in AMPK–ULK1–mTOR pathway activation (131). This creates autophagosome initiation and ultimately leads to autophagic cell death. Notably, caspase inhibition and autophagy inhibition both prevented cell death, suggesting that SIRT6 activation triggers a lethal autophagy response rather than an acute stress response (131). Last, Lv et al. found an interaction between ferroptosis and autophagy in glioblastoma via the SIRT3/NCOA4 pathway (135). Treatment with puerarin induced SIRT3 and increased NCOA4 and LC3-II levels, accelerating ferritin degradation and iron release. This augmented ROS and ferroptotic cell death, whose effect was abolished by autophagy inhibitors or NCOA4 knockdown (135). These data cumulatively validate the double and context-dependent function of SIRTs in autophagy in cancer. While SIRT3, SIRT4, and SIRT6 are poised to induce tumor-suppressive autophagy, others, such as SIRT2 and SIRT7, are likely to stimulate protective autophagic programs that underlie tumor survival and drug resistance. Other evidence that confirms the pro- or anti-autophagic functions of other SIRTs has been listed in **Table 1**, emphasizing the heterogeneity of SIRT-mediated autophagy in cancer.

### 3.3 Glycolysis

The modulation of glycolysis by SIRTs emerges as a pivotal mechanism by which cancer cells reprogram their metabolism to adapt and survive under therapeutic and microenvironmental stress. Among these, SIRT1, SIRT4, and SIRT5 are the most highly implicated regulators, exhibiting both tumor-suppressive and tumor-supportive functions, depending on the cellular and tumor context (**Table 3**). In oxaliplatin-resistant CRC, PARP-driven NAD^+^ depletion reduces SIRT1 levels, thereby enhancing glycolysis via PKM2 and LDHA activation. Restoring SIRT1 reverses this metabolic adaptation and re-sensitizes cells to therapy (136). Zhang and colleagues again demonstrated a specific role for SIRT5 in CRC during hypoxia (137). Through desuccinylation of inorganic pyrophosphatase 2 (PPA2), SIRT5 inhibits its binding to NEDD4, stabilizes hypoxia-inducible factor-1α (HIF-1α), and consequently enhances glycolysis and metastatic capacity during low oxygen levels (137). Conversely, in NK/T-cell lymphoma, SIRT5 functions as a tumor suppressor by desuccinylation and destabilization of glucose-6-phosphate isomerase (GPI), thereby inhibiting glycolytic flux and tumor growth (140).

**Table 3:** Sirtuin-driven modulation of glycolytic pathways and their impact on tumor metabolism and therapy response.

Cancer type	Study type	Glycolysis mechanism	Study conclusion	Ref.
CRC	*In vitro* & *in vivo*	SIRT1 downregulated via PARP → ↑ PKM2/LDHA → oxaliplatin resistance	SIRT1 suppresses glycolysis and reverses drug resistance	(136)
CRC	*In vitro* & *in vivo*	SIRT5 desuccinylates PPA2 → ↑ HIF-1α stability → ↑ glycolysis	SIRT5 stabilizes HIF-1α and enhances glycolysis in hypoxia	(137)
CRC	*In vitro* & *in vivo*	SIRT4 upregulation → HIF-1α degradation → ↓ GLUT1/LDHA → ↓ glycolysis	SIRT4 suppresses glycolysis via HIF-1α degradation	(138)
CRC	*In vitro* & *in vivo*	SIRT1 upregulation → switch glycolysis to FAO via β-catenin deacetylation	SIRT1 mediates glucolipid switch favoring FAO in CRC	(139)
NK/T-cell lymphoma	*In vitro* & *in vivo*	SIRT5 desuccinylates GPI → degradation → ↓ glycolysis	SIRT5 inhibits glycolysis and tumor growth via GPI	(140)
Intestinal cancer	*In vitro*	SIRT4 loss → ↑ glutamine uptake & nucleotide synthesis (indirect glycolysis support)	SIRT4 loss supports metabolic reprogramming post-IR	(141)
Glioma	*In vitro*	Glucose ↓ SIRT1 → ↑ acetyl-HMGB1 → ↑ glioma progression	Glucose downregulates SIRT1, enhancing tumorigenic signals	(142)
Glioma	*In vitro* & *in vivo*	SIRT5 expression → ↓ proliferation, implied ↓ glycolysis	SIRT5 has tumor-suppressive role through metabolic regulation	(85)
Bladder cancer	*In vitro* & *in vivo*	SIRT6 loss → stabilized UHRF1 → ↑ HK2/MCT4 → ↑ glycolysis	SIRT6 loss drives glycolysis and lactate production via UHRF1	(143)
PCa	*In vitro*	Leptosidin ↓ SIRT1/GLUT1/LDHA → apoptosis	SIRT1/GLUT1 inhibition leads to apoptosis	(144)
Lymphoblastoid (TK6)	*In vitro* & *in vivo*	SIRT1 promotes HK2 expression → ↑ glycolysis in long-term HQ exposure	Long-term SIRT1 upregulation promotes glycolysis and tumorigenesis	(145)
Breast cancer	*In vitro*	PGC-1α → ↑ SIRT3 → ↓ glycolysis & ↑ apoptosis	SIRT3 inhibits glycolysis and promotes apoptosis	(146)
Pancreatic cancer	*In vitro* & *in vivo*	SIRT6 upregulation reverses KLF10 loss → ↓ glycolysis via HIF1α/NFκB	SIRT6 suppresses glycolysis and metastasis in PDAC	(147)
Mammary tumor	*In vitro*	SIRT2 deacetylates PKM2 → ↑ tetramerization → ↑ glycolytic flux	SIRT2 promotes glycolysis via PKM2 deacetylation	(148)
Gastric cancer	*In vitro*	SIRT3 deacetylates LDHA → ↑ activity → ↑ glycolysis & proliferation	SIRT3 enhances glycolysis in gastric cancer cells	(149)

A similar tumor suppressor activity is seen for SIRT4 in CRC as well. Zhang et al. demonstrated that sodium butyrate induces the overexpression of SIRT4, which leads to the autophagy-mediated breakdown of HIF-1α, resulting in the repressed expression of GLUT1 and LDHA and, consequently, hindering glucose uptake and lactate synthesis (138). This is corroborated by Tucker et al., who indicated that loss of SIRT4 in intestinal tumors enhances glutamine metabolism and nucleotide synthesis, which indirectly maintains glycolytic intermediates and cell proliferation (141). SIRT1 also has pro-tumorigenic action in prostate and CRC. Park et al. found that SIRT1 increases HK2 expression and mitochondrial binding to enhance glycolysis and tumour development in HQ-treated lymphoblastoid cells (144). Similarly, Wei et al. indicated that SIRT1, under conditions of glucose starvation, enables metabolic adaptation to fatty acid oxidation through the deacetylation of β-catenin, ensuring survival and development in CRC (139).

In glioma, Wang et al. found that high glucose levels suppress SIRT1 activity, leading to the accumulation of acetylated HMGB1 and the activation of a pro-tumorigenic pathway (142). This suggests that loss of SIRT1 under hyperglycemic conditions may contribute to glycolytic reprogramming and the development of malignancy (142). Further evidence for the tumor suppressor function of SIRT5 was demonstrated by Tang et al., who showed that SIRT5 expression in gliomas is associated with decreased proliferation and a better prognosis, most likely through the mitochondrial regulation of metabolism (85). Quantitative measurement of glycolysis was not performed; however, the metabolic shift suggests that the glycolytic function is suppressed (85).

Collectively, these studies highlight the significance of SIRTs as regulators of cancer glycolysis through polyfaceted pathways, including the regulation of key glycolytic enzymes, transcription factors, and metabolic switches. Contextual dependency defines their activity: SIRT1 and SIRT5, for example, suppress or stimulate glycolysis based on the cancer type and metabolic stress. These findings put SIRTs in the limelight as important metabolic regulators and potential therapeutic targets for oncology. Further evidence, consistent with the mechanistic conclusions presented here, is shown in **Table 1**.

## 4 Sirtuins in tumor immunity: a mechanistic overview

SIRTs are central regulators of cellular metabolism, stress resistance, and aging. In the TME, accumulating evidence suggests that SIRTs regulate most features of anti-tumor immunity, including T cell differentiation, natural killer (NK) cell function, immune checkpoint control, and tumor-associated macrophages (TAMs). Here, we summarize the immunological roles of SIRTs in a categorized and concise manner to facilitate a clear understanding of their mechanistic functions.

### 4.1 T Cell differentiation and function

T cell differentiation and function are dynamically and tightly controlled processes that heavily rely on metabolic and epigenetic remodeling within the TME. Among them, NAD^+^-dependent deacetylases, sirtuins, are pivotal in regulating T cell responses, immune evasion, or pro-tumor immunity. SIRT2 expression in peripheral T lymphocytes of breast cancer patients is significantly reduced (19). Their research correlated greater SIRT2 expression with higher CD8^+^ effector memory T (TEM) cell counts and demonstrated that SIRT2 increases aerobic metabolism while suppressing GSK3β acetylation, promoting successful CD8^+^ T cell differentiation and function (19). These observations highlight the therapeutic potential of SIRT2 as a target for enhancing antitumor immunity through T-cell metabolic rewiring (19). In a separate line of inquiry, Hu et al. identified SIRT7 as a metabolic regulator of T cell immunity (20). With Sirt7-knockout mice, they showed that disruption of SIRT7 suppresses BCAA catabolism and enhances fatty acid synthesis, thereby compromising the activation and cytotoxicity of T cells (20). More importantly, they demonstrated that pharmacological modulation of metabolic pathways can restore part of T cell function, and therefore, SIRT7 also acts as a metabolic checkpoint controlling T cell activity within the TME (20).

In parallel, the immunosuppressive functions of other SIRTs have also been uncovered. Zi et al. reported a new SIRT1-CX3CL1 pathway in CRC, which, through SIRT1-mediated release of CX3CL1, enhanced the function and infiltration of regulatory T cells (Treg) (150). Such a switch supported immune evasion by promoting the differentiation of highly suppressive TNFRSF9^+^ Tregs (150). Their results were further confirmed by the *in vivo* effectiveness of CX3CR1 inhibition alongside with anti-PD-1 treatment, indicating a new combinatorial regimen (150). Similarly, Zhang et al. demonstrated that the activation of SIRT6 in cancer cells promoted the differentiation of CD4^+^ T cells into Tregs characterized by increased amounts of immunosuppressive mediators, including adenosine and PD-L1 (151). Transcriptome analysis revealed an oncogenic shift in gene expression; thus, SIRT6 appears to play a role in shaping an immune environment that, although tolerogenic, is neither purely supportive nor detrimental to antitumor immunity (151). In addition to T cells, sirtuin-driven lineage commitment also occurs within the context of overall adaptive immunity. Gamez-Garcia et al. investigated the role of SIRT7 in B lymphopoiesis and showed that SIRT7-facilitated deacetylation of Pax5 serves as an indispensable regulatory step for early B cell development (152). While not directly linked to T cell function, this study emphasizes the broad regulatory scope of SIRTs across immune lineages (152).

Cumulatively, these data place SIRTs as major regulators of T cell activation and differentiation, either enhancing cytotoxicity, as seen with SIRT2 and SIRT7, or conferring immune suppression through Treg expansion mediated by SIRT1 and SIRT6. These bivalently functioning do require careful therapeutic modulation, involving the boosting of immunostimulatory SIRTs and the selective inhibition of immune-suppressive ones. Such treatments can be combined with existing immunotherapies to enhance clinical effectiveness. However, the cell-type- and context-dependent roles of particular SIRTs must be specifically regulated to avoid unwanted immune dysregulation.

### 4.2 T cell anti-tumor immunity

**Figure 4:**
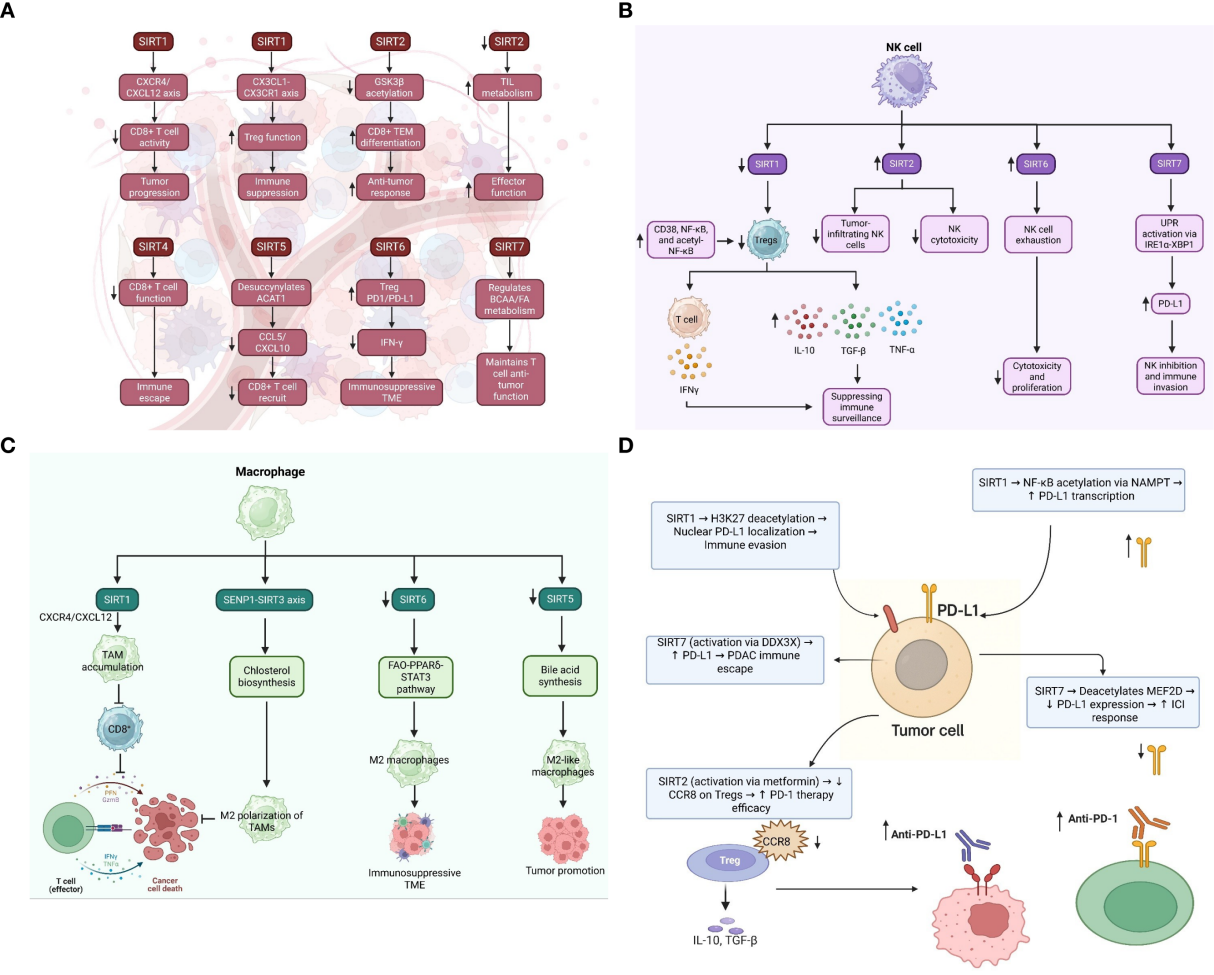
Multifaceted immunoregulatory roles of SIRT1–7 in the tumor microenvironment. **(A)** Sirtuins differentially modulate T cell responses: SIRT1 and SIRT6 promote immunosuppressive signaling and Treg activity, whereas SIRT2 and SIRT7 support cytotoxic T cell function and metabolic fitness. SIRT4 and SIRT5 impair CD8^+^ T cell function and recruitment, contributing to immune escape. **(B)** In NK cells, reduced SIRT1 and SIRT2 limit cytotoxicity and infiltration, while SIRT6 drives NK cell exhaustion and SIRT7 increases PD-L1–mediated immune evasion. **(C)** In macrophages, SIRT1, SIRT3, SIRT4, and SIRT5 regulate TAM polarization toward immunosuppressive M2-like phenotypes through distinct metabolic pathways. In this panel, the green cell represents macrophages/TAMs and the red cell represents tumor cells. **(D)** Sirtuins also modulate immune checkpoint signaling: SIRT1 and SIRT7 regulate PD-L1 expression through acetylation-dependent mechanisms, while SIRT2 activation enhances anti-PD-1 therapy response.

The anti-tumor efficacy of CD8^+^ T cells is profoundly influenced by their metabolic state and the composition of the TME. Recent findings highlight that SIRT enzymes, which depend on NAD^+^, play a crucial role in regulating the function of CD8^+^ T cells, either enhancing or suppressing their immune response based on the specific cellular and molecular environment (**Figure 4A** and **Table 4**). Hamaidi et al. demonstrated that SIRT2 functions as a suppressor of the effector function of CD8^+^ T cells by inhibiting several metabolic pathways, including glycolysis and oxidative phosphorylation (162). The pharmacological inhibition of SIRT2 reprograms TILs to increase their metabolic fitness, proliferative capacity, and anti-tumor function in both mouse and human models (153). Therefore, SIRT2 represents a potential therapeutic target for rejuvenating failing T cells in immunotherapy-resistant tumors. On the other hand, Wan et al. discovered in CRC that the *de novo* generation of NAD^+^ via the kynurenine pathway maintains the function of CD8^+^ T cells by enabling PTEN degradation, thus avoiding metabolic exhaustion (153). The findings show that intrinsic NAD^+^ biosynthesis is subject to regulation to reinvigorate the activity of CD8^+^ T cells in metabolically inhibitory TMEs (153).

**Table 4:** Immunomodulatory roles of sirtuins in shaping tumor immune microenvironments and responses to immunotherapy.

Cancer type	Study type	Involved mechanism	Study conclusion	Ref.
CRC	Experimental (*in vitro*, mouse model, mIHC)	SIRT1 enhances Treg functionality via CX3CL1-CX3CR1 axis and promotes immune suppression	SIRT1-mediated metabolic reprogramming boosts Treg function, aiding CRC progression	(150)
CRC	Experimental (NK cells, flow cytometry)	CD38+ NK cells modulate cytokine secretion via SIRT1/NF-κB pathway	CD38+ NKs suppress immune surveillance; anti-CD38 or SIRT1 activation restores NK function	(21)
CRC	Experimental (human/mouse samples)	*De novo* NAD+ synthesis via kynurenine pathway supports CD8+ T cell metabolic function and antitumor activity	KP-derived NAD+ maintains CD8+ T cell fitness and immune surveillance	(153)
CRC	*In vivo* mouse model, flow cytometry	SIRT6 upregulated in exhausted NK cells; knockdown restores cytotoxicity and proliferation	SIRT6 negatively regulates NK cell function; its inhibition suppresses CRC progression	(154)
CRC	Organoid co-culture, RNA-seq	SIRT1 induces TAM accumulation via CXCR4/CXCL12; inhibits CD8+ T cell function	SIRT1-high CRC leads to T cell suppression and macrophage-driven progression	(155)
HCC	Experimental (mouse model, human samples)	SIRT5 loss promotes bile acid synthesis, induces M2-like TAMs and immunosuppressive TME	SIRT5 prevents immune escape; its downregulation linked to tumor progression via BA signaling	(156)
HCC	*In vitro*/*in vivo* gene therapy, mitochondrial analysis	SIRT3/SIRT4 restore mitochondrial function and reduce malignancy via biogenesis and mitophagy	Co-expression of SIRT3/4 reverses metabolic reprogramming and suppresses tumor progression	(157)
HCC	Mouse models, macrophage polarization	SIRT4 silencing promotes M2 macrophages via FAO-PPARδ-STAT3 axis and MCP-1 induction	SIRT4 downregulation in TAMs enhances HCC development via immunosuppressive shift	(22)
HCC	RNA-seq, *in vitro*, *in vivo* mouse models	CTNNB1 GOF mutation suppresses SIRT2, activates β-catenin/KDM4D complex, increasing MMP9 and immune evasion	SIRT2 downregulation leads to immune suppression; restoring its function improves anti-PD-1 response	(158)
HCC	*In vivo*, gene knockout models	SIRT7 deacetylates MEF2D; represses PD-L1; knockout increases response to PD-1 therapy	SIRT7 suppresses PD-L1 via MEF2D regulation; knockout enhances immune checkpoint efficacy	(23)
Breast Cancer	Experimental (human T cells, mouse model)	SIRT2 promotes CD8+ TEM cells by enhancing oxidative metabolism and reducing GSK3β acetylation	SIRT2 facilitates antitumor immunity via T-cell differentiation and metabolism	(19)
Bladder Cancer	Bioinformatics + Experimental (IMvigor210, TCGA, *in vitro*)	SIRT4 downregulation impairs CD8+ T cell function, promotes tumor growth and immune escape	SIRT4 acts as an immune checkpoint suppressor; its inhibition promotes tumor progression and immune evasion	(159)
Lung Cancer	*In vitro*/*in vivo* mouse models	SIRT2 secreted by macrophages deacetylates extracellular proteins like ITGB3 to promote metastasis	SIRT2 supports cancer cell metastasis via extracellular deacetylation	(160)
Lung Adenocarcinoma (EGFR-mutant)	Molecular assays in LUAD	SIRT5 desuccinylates ACAT1, activates NRF2, reduces CCL5/CXCL10, blocks CD8+ T cell recruitment	SIRT5 promotes suppressive TIME; targetable with ICIs	(161)
Lung Adenocarcinoma	In silico, mRNA/protein profiling	Triacetylresveratrol activates SIRT2, improves immune infiltration, upregulates PD-1	SIRT2 is immunomodulatory in LUAD; potential for combination immunotherapy	(25)
NSCLC	Experimental (TILs, mouse models)	SIRT2 suppresses T cell metabolism; its inhibition enhances TIL fitness and effector function	Pharmacologic inhibition of SIRT2 improves TIL-based immunotherapy	(162)
Melanoma	*In vivo* mouse model, SIRT2 overexpression and inhibition	Systemic SIRT2 overexpression reduces tumor-infiltrating NK cells and impairs NK activity	SIRT2 promotes melanoma progression by inhibiting NK cells; inhibition may support immunotherapy	(163)
Melanoma	Spatial proteomics, mouse models	SIRT1 correlates with CD8+ T cell infiltration and enhances response to PD-1 therapy	SIRT1 promotes T-cell infiltration and improves ICI response in melanoma	(164)
Melanoma	*In vitro*/*in vivo*, molecular studies	SIRT7 activates UPR via IRE1α-XBP1, promotes PD-L1 and immune evasion, suppresses SMAD4	SIRT7 promotes immune evasion and is a therapeutic target to enhance PD-1 blockade	(165)
Melanoma (Pan-Cancer)	Pan-cancer bioinformatics + experiments	SIRT5 expression correlates with immune infiltration and immunotherapy biomarkers	SIRT5 predicts prognosis and immunotherapy response; key target in melanoma	(166)
Ovarian Cancer	EV analysis, mouse models	SIRT1 in CAA-derived EVs activates CD24/Siglec-10 axis, promotes CD8+ T cell apoptosis	SIRT1-EVs suppress immune response and promote tumor growth	(167)
Cervical Cancer	Proteomics, transcriptomics, functional analysis	NAMPT-SIRT1 axis deacetylates H3K27, induces PD-L1 nuclear localization, promotes immune evasion	SIRT1 modulates PD-L1 via epigenetics; NAMPT-SIRT1 axis key in resistance to PD-1 blockade	(24)
GBM	Mouse model, T cell assays	Metformin activates AMPK–SIRT2, reduces CCR8 expression on Tregs, enhances PD-1 therapy	SIRT2 activation via metformin improves immune response and anti-PD-1 efficacy	(168)
Glioma	Bioinformatics analysis (TCGA/CGGA datasets)	NAD+ metabolism gene signature including SIRT-related genes affects immune microenvironment and therapy response	Prognostic NAD+ metabolism signature predicts ICI response; SIRTs linked to immunosuppression	(169)
Pancreatic Ductal Adenocarcinoma	*In vitro*/*in vivo* molecular biology	DDX3X binds SIRT7 to induce PD-L1 and tumor progression	DDX3X-SIRT7 axis promotes immune evasion and PDAC growth; SIRT7 is therapeutic target	(170)
B-cell Development & B-ALL	Experimental (*in vitro*, mouse model)	SIRT7 deacetylates Pax5 at K198, stabilizing it and guiding B cell lineage and function	SIRT7 essential for B-cell development; its loss disrupts lineage commitment	(152)
Pan-Cancer	Bioinformatics + *in vivo*	NAMPT/SIRT-mediated acetylation of NF-κB p65 regulates PD-L1 transcription; dual inhibitor developed	NAMPT and PD-L1 co-targeting enhances antitumor immunity and T cell activation	(171)
Pan-Cancer	Bioinformatics + Experimental Validation	SIRT1 expression correlates with immune cell infiltration and immune regulation in various cancers	SIRT1 plays a role in immune infiltration and is a candidate for targeted cancer immunotherapy	(172)
Multiple tumor cell lines (e.g., HeLa, SW480)	Experimental (*in vitro*, transcriptomics)	SIRT6 activation increases Tregs, PD-1/PD-L1, ADO, and decreases IFN-γ, promoting immunosuppressive TME	SIRT6 enhances immune evasion by reprogramming TME and suppressing immune surveillance	(151)
General (T-cell immunity)	Experimental (mouse model, proteomics)	SIRT7 regulates BCAA/FA metabolism, maintaining T-cell function and preventing exhaustion	SIRT7 deficiency impairs T-cell antitumor function via metabolic disruption	(20)
General (human NK cells)	CRISPR knockout, *in vitro* and *in vivo* validation	SMAD4 knockout enhances NK resistance to TGFβ/activin A; improves cytotoxicity and proliferation	SMAD4KO boosts NK antitumor activity and therapy response across NK cell platforms	(173)
Tumor-associated Macrophages (various cancers)	Experimental (metabolomics, flow cytometry)	SENP1-SIRT3 axis drives cholesterol synthesis, leading to M2 macrophage polarization	SIRT3 promotes immunosuppressive TAMs and T cell inhibition; potential target for therapy	(174)

SIRT5 is also involved in modulating immune suppression in various types of tumor cancers. Shouhan et al. demonstrated that in epidermal growth factor receptor (EGFR)-mutated lung adenocarcinoma (LUAD), succinylation of ACAT1 by SIRT5 activates the nuclear factor erythroid 2–related factor 2 (Nrf2) pathway, which suppresses chemokine secretion essential for CD8^+^ T cell recruitment (161). This highlights the cell type- and context-dependent duality of SIRT5, which promotes anti-tumor immunity in certain contexts and immune evasion in others (161). Likewise, Lv et al. demonstrated a tumor-suppressive function for SIRT4 in bladder cancer (159). Their findings indicate that SIRT4 maintains CD8^+^ T cell chemotaxis and cytotoxic activity, and that inhibition of SIRT4 impairs T cell function, promoting tumor immune escape (159). These results provide mechanistic insight into how mitochondrial metabolism, regulated by sirtuins such as SIRT4, affects T-cell-tumor interactions.

In HCC, an immunosuppressive microenvironment caused by SIRT5 Deficiency was linked to augmented bile acid biosynthesis, resulting in M2-like macrophage polarization and defective CD8^+^ T cell surveillance (156). Based on evidence presented by Cai et al., inhibiting bile acid signaling with drugs such as cholestyramine was able to rescue this suppressive effect, suggesting that metabolic crosstalk between hepatocytes and immune cells is the major determinant of CD8^+^ T cell function (156). In addition, in CTNNB1-mutant HCC, SIRT2 inhibition indirectly enhances MMP9 expression, which inhibits CD8^+^ T cell infiltration and signaling. MMP9 inhibition remodels the TME and makes tumors responsive to anti-PD-1 therapy again, as shown by Gut et al. (158). These findings highlight how sirtuin modulation can contribute to checkpoint blockade in cancers that are otherwise resistant.

Collectively, these studies suggest that SIRTs are key regulators of CD8+ T cell function, which is disrupted by both direct metabolic remodeling and indirect remodeling of the TME. Therapeutic approaches involving the selective modulation of SIRT activity are likely to enhance CD8+ T cell responses, either by inhibition—for example, SIRT2 and SIRT5 in LUAD, or by activation—for instance, SIRT4 in bladder cancer—to overcome resistance to immunotherapy. Of note, their dual functions between cancer types call for rigorous targeting strategies and biomarker-based patient selection to avoid counterproductive effects.

### 4.3 NK cell suppression

NK cells are crucial elements of innate immune regulation and play a critical role in controlling early tumor growth. Yet, in the TME, several mechanisms exist that inhibit NK cell cytotoxicity, facilitating immune evasion and disease progression. Increasing numbers of studies have begun to uncover the distinct mechanisms implicated in this suppression, including sirtuins, TGF-β signaling, and transcriptional regulators. Systemic overexpression of SIRT2 accelerates melanoma growth and reduces NK cell infiltration, as well as impairing NK cell cytotoxicity (163). Pharmacologic SIRT2 inhibition antagonized the effect, increasing NK cell activity and infiltration (**Figure 4B** and **Table 4**) (163). These findings suggest that SIRT2 is an immunosuppressive regulator of melanoma, directly suppressing NK cell function (163). Therapeutically, SIRT2 would be an attractive target for augmenting innate antitumor immunity, particularly in cancer types where NK cell dysfunction is an etiopathogenic factor leading to immune escape. Similarly, for SIRT6 in CRC, the same held good. In a mouse model of inflammatory CRC, SIRT6 was elevated in NK cells that had an exhausted phenotype (154). Knockdown of SIRT6 promoted NK cell proliferation and the production of cytotoxic mediators, and the adoptive transfer of SIRT6-null NK cells inhibited tumor growth (154). These results from the SIRT6 study highlight the role of this sirtuin in NK cell exhaustion and demonstrate that it acts as a negative regulator of NK-mediated tumor surveillance.

In a similar process, SIRT1 activity was found to be lower in CD38^+^ NK cells from CRC patients, resulting in higher NF-κB acetylation and the release of immune-suppressing substances, such as IL-10 and TGF-β (21). Wang et al. showed that this biased cytokine profile enhanced Treg differentiation and M2 macrophage polarization to enhance a suppressive TME (21). SIRT1 activation or inhibition of CD38 or NF-κB reversed the impact and restored a pro-immunity cytokine milieu (21). Rea et al. used gene editing to reverse NK suppression by blocking the TGFβ/activin A-SMAD4 pathway (173). CRISPR-Cas9 was used to delete SMAD4 in human NK cells, rendering them unresponsive to TGFβ and activin A signals. *In vivo*, SMAD4KO NK cells retained their cytotoxic and cytokine reactivity to immunosuppressive signals while also exhibiting increased tumor infiltration and tumor suppression (173). Gene-engineered NK cells outperformed pharmaceutical TGFβ suppression and were effective across numerous NK cell platforms, including CAR-NK and stem-cell-derived NK cells (173). Overall, these findings show that there are many ways NK cell activity is reduced in cancer, influenced by epigenetic enzymes like SIRTs (SIRT1, SIRT2, SIRT6), surface regulators like CD38, and signaling pathways like TGFβ-SMAD4. Although each pathway plays a unique role in NK dysfunction, they all contribute to the suppression of cytotoxicity, cytokine signaling, and proliferation. A promising approach to boost NK cell ability to fight tumors is to combine gene editing (e.g., knocking out SMAD4) with changing their metabolism (e.g., inhibiting or activating sirtuins). Future studies should focus on context-specific interventions that precisely restore NK function without inducing off-target immune activation.

### 4.4 Dendritic cells: glycolytic maturation and SIRT-dependent immunogenic tuning

Dendritic cells (DCs) are key modulators of anticancer immunity (175, 176), and their development necessitates a metabolic shift toward increased glycolysis (177, 178). Within this immunometabolic context, sirtuins act as key modulators of DC activation, cytokine output, and T-cell priming. SIRT1 suppresses DC activation by deacetylating NF-κB p65, thereby reducing the expression of co-stimulatory molecules and IL-12 secretion (179), which in turn weakens CD8^+^ T cell priming (179). Moreover, SIRT1 links DC metabolism to tolerogenic versus inflammatory programming. In obesity, SIRT1 activity is reduced in Bone marrow-derived dendritic cells (DCs), with elevated extracellular acidification rates (ECAR)/oxidative phosphorylation (OXPHOS), increased Major histocompatibility complex class II (MHCII)/CD86/CD40, higher IL-12p40 and lower TGF-β, coincident with suppression of the IDO1–kynurenine pathway; mechanistically, SIRT1 positively regulates Ido1 in a PPARγ-dependent manner, positioning SIRT1 as a gatekeeper of tryptophan catabolism and T-cell polarization (180). Independently, a DC-intrinsic SIRT1–hypoxia-inducible factor 1-alpha (HIF-1α) checkpoint programs reciprocal IL-12 versus TGF-β1 output to bias naïve CD4^+^ T cells toward TH1 rather than Treg fate, establishing SIRT1 as a determinant of DC-guided T-cell lineage specification (181).

SIRT1 plays a role in antiviral immunity. DCs upregulate SIRT1 during respiratory syncytial virus infection to promote autophagy-associated activation and the production of antiviral cytokines. The inhibition of SIRT1 by genetic or pharmacological means results in impaired DC activation and worsens disease pathology (182). In addition to SIRT1, pathogens can also utilize SIRT2 to suppress host immune responses. For example, *Salmonella* increases SIRT2 levels in dendritic cells, which promotes NF-κB-dependent nitric oxide synthase-2 (NOS2) expression and elevates nitric oxide production. This sequence ultimately restrains T-cell proliferation. Blocking SIRT2 counteracts these effects and lowers the bacterial load *in vivo*, suggesting that SIRT2 could serve as a potential target for host-directed immunotherapy (183). At the same time, SIRT6 is required for proper DC development and function; Sirt6 deficiency reduces the frequency of conventional DC precursors, diminishes the expression of co-stimulatory molecules and MHC-II, impairs C-C chemokine receptor type 7 (CCR7)-mediated migration, and weakens T-cell activation, effects also observed in human monocyte-derived DCs following SIRT6 inhibition (184).

Further indications of this regulatory paradigm can also be found in other antigen-presenting cells. For instance, SIRT1 is required in B cells to enable effective MHC-II antigen presentation and to support proper activation of CD4^+^ T cells (185). In a different context, mitochondrial SIRT4 influences the transition of monocytes between glycolytic and oxidative metabolic states; through this adjustment, it helps to reverse immune tolerance and restore their ability to mount inflammatory responses (186). When considered together, these observations suggest a broader organizing principle: sirtuins appear to link cellular metabolic conditions with the efficiency of antigen presentation and with the overall immunogenic character of DCs. Taken together, these observations suggest that sirtuins play a central role in shaping dendritic cell metabolism and, as a result, help determine whether immune responses lean toward Type 1 T helper (Th1) activation or a more tolerogenic, Treg-favoring profile. Since dendritic cells initiate and maintain anti-tumor T-cell activity, shifts in their function caused by SIRT signaling are likely to affect both the strength of the anti-cancer response and its overall character. In this way, adjusting SIRT activity in DCs may offer a practical and biologically grounded strategy for influencing immune behavior within the TME.

### 4.5 Tumor-associated neutrophils and PMN-MDSCs: SIRT-dependent suppressive and angiogenic programming

Myeloid-derived suppressor cells (MDSCs) are immature myeloid populations with strong immunosuppressive activity in the tumor TME. They comprise two principal subsets: polymorphonuclear MDSCs (PMN-MDSCs) and monocytic MDSCs (M-MDSCs), both of which help establish a tumor-promoting, immunosuppressive niche (187). Neutrophils have also been increasingly recognized as key players in the progression of tumors. In the context of the TME, tumor-associated neutrophils (TANs) exhibit a high degree of plasticity and can adopt either an anti-tumor or pro-tumor role, shaped mainly by the signals present in their immediate environment (188). In their anti-tumor mode, neutrophils can directly kill malignant cells and enhance adaptive immune priming; in their pro-tumor mode, TANs promote disease progression by fostering angiogenesis, supporting tumor proliferation, and suppressing cytotoxic immune activity (188). A representative immunosuppressive mechanism involves TAN-mediated apoptosis of non-activated CD8^+^ T cells through TNFα- and nitric-oxide–dependent pathways, which narrows the pool of functional cytotoxic T cells and favors a tumor-permissive immune milieu (189). This selective depletion further strengthens the immune-evasive conditions shaped by MDSCs and by metabolically reprogrammed TANs.

Recent work has shown that sirtuins sit upstream of key transcriptional and metabolic programs that shape the behavior of both TANs and PMN-MDSCs. In tumors where interferon signaling is weak, TANs tend to shift toward a pro-angiogenic profile. Under these circumstances, TANs increase their production of vascular endothelial growth factor (VEGF), matrix metalloproteinase-9 (MMP-9), and BV8 (also known as prokineticin 2). This response depends on SIRT1, which deacetylates FOXO3a and helps retain it within the nucleus, where it can activate genes related to angiogenesis. When SIRT1 is absent or repressed, FOXO3a fails to stay nuclear, resulting in decreased expression of these angiogenic factors. Therefore, SIRT1 functions as an important regulator of the angiogenic profile of TANs (190). In PMN-MDSCs, however, the role of SIRT1 is different. Here, SIRT1 helps maintain their immunosuppressive character. Loss of SIRT1 in these cells activates an mammalian target of rapamycin (mTOR)–HIF-1α pathway that drives a shift toward an M1-like state. These cells then show less suppressive activity and gain tumor-killing capacity. In other words, SIRT1 is necessary to maintain the suppressive identity of MDSCs, and when it is inhibited, the balance shifts toward a more inflammatory and tumor-rejection-oriented immune response (191).

In a sarcoma model undergoing stem cell transplantation, removing granulocytic MDSCs led to noticeably smaller tumors, along with an apparent increase in T-cell infiltration into the tumor area. Interestingly, this situation was also linked to a marked reduction in SIRT1 expression within the tumor tissue itself (192). This indicates that when SIRT1 activity is low, PMN-MDSCs are less capable of maintaining their suppressive state, thereby allowing T cells to be more effective against the tumor. In other words, SIRT1 contributes to the suppressive character of these cells, and when it is diminished, the balance flips back to anti-tumor immunity (192). Taking all of this together, SIRTs appear to act as upstream metabolic and transcriptional regulators that influence whether neutrophil-lineage cells participate in immune evasion or, instead, assist cytotoxic responses against tumors. On this basis, targeting SIRT-controlled pathways in TANs and PMN-MDSCs could provide a practical approach to restore CD8^+^ T-cell function and potentially enhance the effectiveness of anti-cancer therapy.

### 4.6 Remodeling of the TME

Tumor immune microenvironment (TIME) determines the direction of malignancy growth or regression by regulating immune cell infiltration, phenotype, and function (193–196). Recent evidence has elucidated how SIRTs and metabolic networks regulate immune remodeling, specifically macrophage polarization, T cell function, and stromal signaling (**Figure 4B**, **Table 4**). One of the pioneering works by Li et al. demonstrated that SIRT4 inhibition in TAMs promoted HCC initiation by shifting macrophage polarization towards the M2 type through the fatty acid oxidation (FAO)–PPARδ–Signal transducer and activator of transcription 3 (STAT3) pathway (22). This reprogramming was associated with increased MCP-1 production, thus recruiting macrophages and promoting immunosuppression (22). This therefore supports the hypothesis that metabolic reprogramming of TAMs could be one critical determinant that orchestrates the tumor immune microenvironment (TIME) toward tumor suppression. Similarly, SIRT1 has also been a double-edged sword in the context of TIME. In CRC, Fang et al. demonstrated that the overexpression of SIRT1 in tumor cells increased CXCL12, facilitating the recruitment of CXCR4^+^ monocytes and thereby promoting TAM accumulation (155). The recruited TAMs suppressed CD8^+^ T cell proliferation, thereby compromising antitumor immunity (155). This process demonstrates how the overexpression of SIRT1 in tumors facilitates an environment that suppresses the immune response.

In ovarian cancer, Zheng et al. demonstrated that cancer-associated adipocyte-derived EVs delivered SIRT1 to tumor cells and stimulated the CD24/Siglec-10 axis (167). This induced CD8^+^ T cell apoptosis, thereby facilitating immune evasion and highlighting the role of stromal-derived SIRT1 in promoting immune remodeling in various types of tumors (167). From a metabolic angle, the SENP1–SIRT3 axis was found to drive cholesterol biosynthesis in TAMs, promoting M2 polarization and suppressing CD8^+^ T cell responses (174). These findings emphasize that metabolic reprogramming is not just a hallmark of cancer cells, but also of the immune cells that populate the TME (174). Targeting these metabolic switches could serve to reawaken anti-tumor immunity in immunologically “cold” tumors (174).

A more clinical view is presented in the work by Placke et al., where SIRT1 activity was found to be linked to CD8^+^ T cell invasion and response to immune checkpoint inhibitor therapy in melanoma (164). Spatial proteomics revealed that SIRT1 is enriched in CD8^+^-high compartments, and pharmacological activation of SIRT1 increased α-PD-1 potency by enhancing pro-inflammatory chemokines, such as CXCL9 and IFN-γ (164). This shift from previous research suggests a tissue-specific and context-dependent immunosuppressive role of SIRT1 in CRC and ovarian cancer, but an immune-enhancing role in melanoma (164). Finally, Zhang et al. demonstrated that bialternative expression of SIRT3 and SIRT4 restored the mitochondrial function and induced differentiation of HCC cells (157). This not only inhibited tumor growth but also regulated the tumor microenvironment by potentially reducing the metabolic signals responsible for immune evasion (157). Overall, TIME remodeling is integrally connected to the metabolic and epigenetic status of cancer and immune cells. Sirtuins, including SIRT1, SIRT3, and SIRT4, are master regulators that can exert either an activating or inhibitory effect on immune activation, depending on the cellular context and cancer type. While some SIRTs form barriers to immune cell penetration and activation, others are promising for promoting responsiveness to immunotherapy. Therefore, accurate mapping of the TIME and sirtuin patient-specific profiles may be the key to unlocking combinatorial therapies targeting single mechanisms.

### 4.7 Integrated cross-isoform regulatory network of SIRTs in tumor immunity

Across malignancies, the immunological consequences of sirtuin expression do not derive from isolated isoform function but rather from a cross-isoform regulatory network in which SIRTs operate as cooperative or antagonistic modules that shape the tumor TME. Converging evidence indicates that SIRT1, SIRT6, and SIRT5 predominantly drive immune-suppressive programs, including regulatory T cell expansion, metabolic suppression of CD8^+^ effector cells, and reinforcement of checkpoint signaling, whereas SIRT2 and SIRT4 support cytotoxic immunity by sustaining effector metabolism and chemokine-guided CD8^+^ infiltration (19, 150, 151, 153, 156, 159, 161, 162). The immune phenotype of the tumor, whether immune-inflamed (“hot”) or immune-excluded (“cold”) (197, 198), thus emerges from the relative dominance of these opposing sirtuin-driven regulatory axes, rather than the expression level of any single SIRT isoform.

SIRT6 serves as a crucial immunosuppressive node in various types of tumors. Recent evidence shows that increasing SIRT6 activity in tumor cells directly drives naive CD4^+^ T-cell differentiation toward regulatory T cells, elevates PD-1 expression in CD4^+^ T cells, and simultaneously upregulates PD-L1 and adenosine production in tumor cells, while reducing IFN-γ and other pro-inflammatory cytokines (151). This establishes SIRT6 as a tumor-intrinsic inducer of immune tolerance, not limited to a single cancer, but conserved across ovarian, cervical, hepatocellular, breast, and colorectal tumor settings (151). In addition, SIRT1 supports immune escape through the facilitation of Treg infiltration and metabolic suppression of effector T-cell function, further reinforcing a tolerogenic TME (150). In EGFR-mutant lung adenocarcinoma, SIRT5 suppresses CD8^+^ T-cell recruitment through an ACAT1–NRF2 succinylation axis, providing an additional layer of suppression that limits responsiveness to checkpoint blockade (161). In HCC, loss of SIRT5 results in M2 macrophage polarization and impaired CD8^+^ surveillance, whereas the inhibition of bile-acid signaling restores immune responsiveness and improves anti-PD-1 efficacy (156, 158).

By contrast, SIRT2 and SIRT4 support cytotoxic anti-tumor immunity. SIRT2 enhances the metabolic fitness and effector differentiation of CD8^+^ T cells, promoting durable anti-tumor responses in breast cancer and glioma models (19, 162). In bladder cancer, SIRT4 regulates the chemotaxis and cytotoxicity of CD8^+^ T cells, whereas its inhibition leads to immune evasion and tumor development (159). These isoforms, therefore, act as counterweights opposing the immunosuppressive activities of SIRT1/SIRT6/SIRT5.

Isoform antagonism is especially pronounced in the context of melanoma, as SIRT1 associates with chemokine-rich CD8^+^-high niches and reinforces responses to PD-1 blockade, while SIRT7 triggers the IRE1α–XBP1 stress pathway to upregulate PD-L1 and promote immune evasion (164, 165). Furthermore, the ability of SIRT7 to both repress and enhance PD-L1 expression, depending on the tumor lineage (23, 189), underscores its bi-functional nature within this regulatory system.

**Figure 5:**
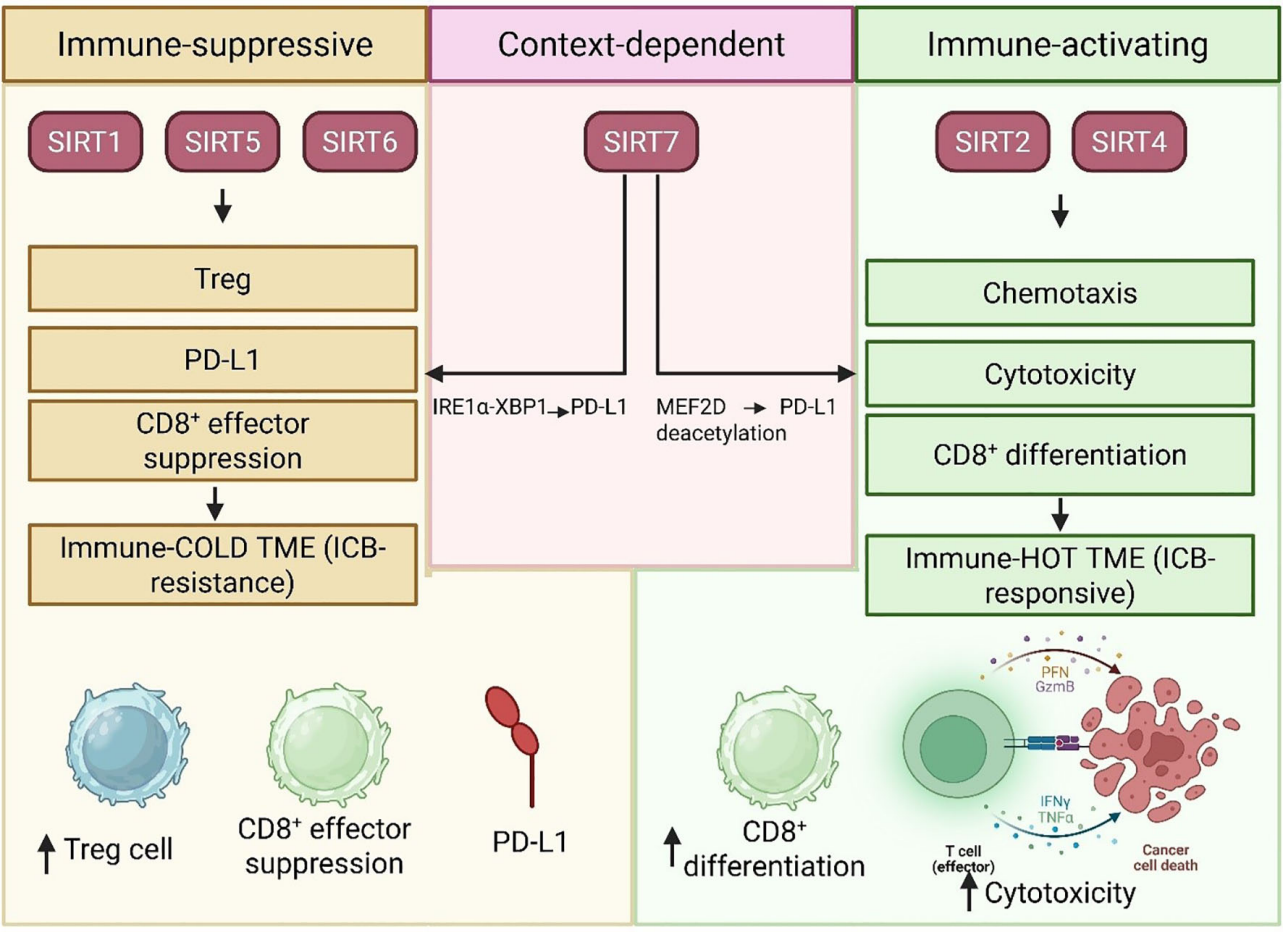
Isoform-specific sirtuin axes define immune-cold versus immune-hot tumor microenvironments. SIRT1, SIRT5, and SIRT6 form an immune-suppressive axis that enhances Treg activity and PD-L1 expression while suppressing CD8^+^ effector function, leading to an immune-cold, ICB-resistant TME. In contrast, SIRT2 and SIRT4 create an immune-activating axis that promotes CD8^+^ T cell chemotaxis, differentiation, and cytotoxicity, supporting an immune-hot, ICB-responsive TME. SIRT7 displays context-dependent behavior: activation of the IRE1α–XBP1 pathway increases PD-L1 and aligns with immune suppression, whereas MEF2D deacetylation reduces PD-L1 and favors immune activation. The overall immune phenotype reflects the balance between these opposing sirtuin-driven programs.

In summary, this integrated model positions SIRT1/SIRT6/SIRT5 as an immune-suppressive sirtuin axis that drives Treg expansion, PD-L1 expression, adenosine accumulation, macrophage skewing, and CD8^+^ exclusion, while SIRT2/SIRT4 constitute an immune-activating axis that sustains effector CD8^+^ differentiation, cytotoxic infiltration, and maintenance of anti-tumor immunity (**Figure 5**, **Table 5**). The ultimate immune landscape of the tumor, immune-hot versus immune-cold, is thus determined by the balance between these contrasting isoform clusters. This framework gives a mechanistic rationale to isoform-targeted therapeutic strategies that are designed to shift the TME from immune-suppressive to immune-responsive states with a view to enhancing the effectiveness of immunotherapy.

**Table 5:** Integrated cross-isoform regulatory network of SIRTs across cancers and immune outcomes.

Isoform(s)	Cancer context(s)	Primary immune axis impacted	Key mechanism(s)	Direction of effect	Net TME outcome	Translational implication	Ref.
SIRT1 + SIRT6	Multiple solid tumors	Treg ↑, PD-L1 ↑, CD8^+^ effector suppression	Tumor-intrinsic SIRT1/SIRT6 promote Treg differentiation & checkpoint signaling	Immunosuppressive	Immune-cold, tolerance-prone	Consider targeted inhibition ± anti-PD-1	(151)
SIRT5	EGFR-mutant LUAD	CD8^+^ recruitment ↓	ACAT1–NRF2 succinylation pathway suppresses chemokines	Immunosuppressive	CD8^+^ excluded microenvironment	Combine SIRT5 inhibition with ICB	(161)
SIRT4	Bladder Cancer	CD8^+^ chemotaxis & cytotoxicity ↑	Mitochondrial regulation sustains T cell effector state	Pro-immunity	Immune-hot	SIRT4 activation may enhance ICB outcome	(159)
SIRT2	Breast Cancer, Glioma T cells	Effector/memory CD8^+^ differentiation ↑	Supports glycolysis + OXPHOS in T cells	Pro-immunity	Sustained antitumor immunity	SIRT2 activation as PD-1 co-adjuvant	(162)
SIRT7	Melanoma/HCC/PDAC	PD-L1 regulation and T-cell suppression	Melanoma: SIRT7 activates IRE1α–XBP1 stress signaling to upregulate PD-L1 and induce immune evasion. HCC: SIRT7 deacetylates MEF2D to suppress PD-L1. PDAC: SIRT7 cooperates with DDX3X to increase PD-L1.	Context-dependent (immune-suppressive polarity in melanoma & PDAC/variable in HCC)	Determines immune-hot vs immune-cold phenotypic outcome based on lineage & stress signaling state	Requires lineage-aware targeting strategy; SIRT7 inhibition + anti-PD-1 may restore checkpoint responsiveness, especially in melanoma	(23, 165, 170)

## 5 Therapeutic implications

Emerging evidence has broadened our understanding of how NAD^+^ metabolism and sirtuin pathway activity influence immune response and tumor growth, uncovering actionable targets for new cancer therapies. These pathways not only control cancer cell survival but also rearrange the immune landscape within the tumor, directly impacting the efficacy of immunotherapy. Yi et al. found that SIRT7 promotes melanoma growth via activating the IRE1α-XBP1 pathway, leading to increased survival and immune evasion of tumor cells (165). Mechanistically, SIRT7 orchestrates tumor progression and immune evasion by selectively activating the UPR. Specifically, SIRT7 deacetylates SMAD4, relieving its transcriptional repression on IRE1α, which in turn activates the IRE1α–XBP1 signaling axis. This activation triggers downstream ERK pathway signaling and secretion of pro-tumor cytokines, supporting cell survival under stress. Importantly, activation of this axis also upregulates PD-L1 expression, thereby facilitating immune escape and resistance to immune checkpoint blockade in melanoma (165). Consistently, PD-L1 has also been reported to regulate metastatic proliferation in NSCLC through the same IRE1α–XBP1 pathway in TAMs (199). IRE1α inhibition decreased PD-L1 expression, suppressed tumor migration and invasion, and improved the treatment outcomes of nivolumab. Importantly, overexpression of SIRT7 drove PD-L1 induction and caused resistance to immune checkpoint blockade (165). A combination of SIRT7 inhibition with anti-PD-1 therapy synergistically enhanced tumor control *in vivo*, which implied that SIRT7 inhibition enhances checkpoint immunotherapy (165). These findings identify SIRT7 as a dual-modality target interfering with tumor cell stress responses and the immune interface. Similarly, in glioma, the development of an NAD^+^ metabolism-related gene signature (NMRGS) yielded a robust biomarker for stratifying patient responses to immune checkpoint inhibitors (ICIs) (169). High NMRGS score patients presented with a more suppressive TME, but also exhibited increased TMB and HLA expression, and were therefore more amenable to ICI treatment (169). This demonstrates the predictive as well as the prognostic significance of NAD^+^-associated gene signatures in relation to the direction of individualized immunotherapeutic protocols.

Mechanistically, Wu et al. revealed that extracellular SIRT2 (eSIRT2) has a specific function in enhancing the metastasis of lung cancer (160). Secreted from TLR-activated macrophages, eSIRT2 deacetylates the TME integrin β3 and enhances cell migration. Although immunologically not immediately related to immune checkpoint therapy, such discoveries suggest that inhibition of eSIRT2 activity can retard metastasis and enhance therapeutic effectiveness, particularly when combined with immune-based strategies targeting metastatic sites (160). At the pan-cancer level, SIRT1 analysis revealed that SIRT1 has a bivalent function, involved in DNA repair, inflammation, and immune infiltration (172). Although SIRT1 was downregulated in various cancers, SIRT1 expression was associated with different immune cells, such as Th2 cells and memory T cells, indicating that modulation of SIRT1 activity would reprogram the immune landscape in the tumor (172). The interaction of SIRT1 with immunomodulatory compounds in most forms of cancer ensures it is a target for immuno-metabolic therapy approaches (172). Together, these investigations position SIRTs and NAD^+^ metabolism at the intersection of immune regulation biology and cancer cell biology. Therapeutically, SIRT7 or secreted SIRT2 are potential targets for suppressing tumor growth and stimulating immune response. Concurrently, the prognostic value of NAD^+^-related gene expression signatures and SIRT1 expression profiles underscores the growing relevance of personalized therapy in light of metabolic-immune interactions. Next-generation therapeutic strategies will be supported by the convergence of sirtuin inhibition and immune checkpoint therapies to overcome resistance and enhance patient outcomes.

## 6 Mechanisms of SIRTs in immunotherapy

Recent findings indicate that various members of the SIRT family play multiple roles in regulating immune checkpoint molecules, the migration of immune cells, and modulating tumor metabolism to either support or inhibit anti-tumor immunity (**Figure 4C**, **Table 4**). Lu et al. demonstrated that SIRT1, activated by the NAD^+^ salvage enzyme NAMPT, induces histone H3K27 deacetylation, which further regulates nuclear retention and PD-L1 expression in cervical cancer cells. This NAMPT/SIRT1 pathway not only reinitiates epigenetic control of immune checkpoint proteins but also presents a mechanistic explanation for PD-1/PD-L1 blockade resistance (24). This regulatory pathway links changes in metabolism to the ability of cancer cells to avoid the immune system, creating an opportunity to improve responses to immunotherapy (24). SIRT2 was identified by He et al. as a candidate immune-supportive factor in lung adenocarcinoma (25). Their findings linked higher levels of SIRT2 to better survival rates in patients and increased presence of cytotoxic and memory T cells. Triacetylresveratrol, a SIRT2-selective activator, has become a promising co-treatment for PD-1-based therapies (25). Building on this pathway, Li et al. reported that metformin-induced activation of SIRT2 inhibits glioblastoma CCR8 expression, thereby reducing the frequency of Treg cells and enhancing T cell-dependent tumor clearance (168). These observations make SIRT2 a significant metabolic–immune checkpoint of bivalency, crucial for tumor regulation and the promotion of immunotherapy.

Rather, Shouhan et al. demonstrated that SIRT5 drives immune evasion in EGFR-mutant lung adenocarcinomas by desuccinylating ACAT1, activating NRF2, and suppressing the release of chemokines essential for CD8^+^ T cell recruitment (161). This desuccinylation network suppresses the immunogenicity of the TME, rendering tumors immunologically cold to ICB. In particular, a pan-cancer meta-analysis corroborated by Ji et al. highlights the prognostic value of SIRT5, attributing it to heterologous immunomodulatory signatures and discordant immunotherapy responses (166). These context-dependent functionalities emphasize the multifaceted role of SIRT5 as both a candidate biomarker and therapeutic target.

SIRT6, identified by Zhang et al., is a highly immunosuppressive program that skews CD4^+^ T cell differentiation towards Treg (151). When SIRT6 is activated, it leads to higher levels of PD-1 and PD-L1, increases adenosine production, and reduces the production of pro-inflammatory cytokines (151). These combined alterations compromise immune surveillance, suggesting that increased SIRT6 activity may be a mechanism of intrinsic resistance to checkpoint inhibitors. SIRT7’s regulatory role is uniquely dualistic. In HCC, Xiang et al. identified that SIRT7 suppresses PD-L1 expression by deacetylating MEF2D, thereby restricting immune suppression (23). On the other hand, in pancreatic ductal adenocarcinoma, Zhao et al. found that SIRT7, working in conjunction with DDX3X, increases PD-L1 expression and facilitates the progression of cancer cells (170). This duality reflects the context-dependent nature of SIRT7 signaling, which is likely influenced by the tumor lineage and interactions with co-regulatory proteins. Finally, Yang et al. demonstrated that NAMPT and PD-L1 interact with each other, partly through SIRT, influencing the acetylation of NF-κB p65 (171). Their dual inhibitor, LZFPN-90, which targets both NAMPT and PD-L1, was shown to strongly activate T cells and combat tumors *in vivo*, highlighting the promise of targeting both metabolic and immune checkpoints simultaneously (171). Collectively, these studies depict an advanced landscape where SIRTs are molecular rheostats of immunogenicity, causing activation or facilitating escape. Cellular metabolism, epigenetic control, and checkpoint regulation are intricately intertwined in their activities, making them both attractive and challenging targets for the development of next-generation immunotherapeutic strategies. The future challenge is to dissect the tissue-specificity and contextuality of each member of the SIRT families to guide precision immunotherapy.

## 7 Mechanistic roles of sirtuins in chemotherapy resistance and sensitization

SIRTs have emerged as critical regulators in shaping cellular responses to chemotherapeutic agents. Their activity regulates multiple biological processes, including metabolism, autophagy, redox state, DNA repair, and immune regulation, which play crucial roles in determining chemotherapy resistance and sensitivity. This chapter carefully delineates how different sirtuin isoforms regulate these processes in various cancers and treatments. To ensure that each subsection is as concise and science-independent as possible, it will describe a specific cellular process or functional pathway through which SIRTs modulate the effectiveness of chemotherapy.

### 7.1 Mechanisms promoting chemoresistance

**Figure 6:**
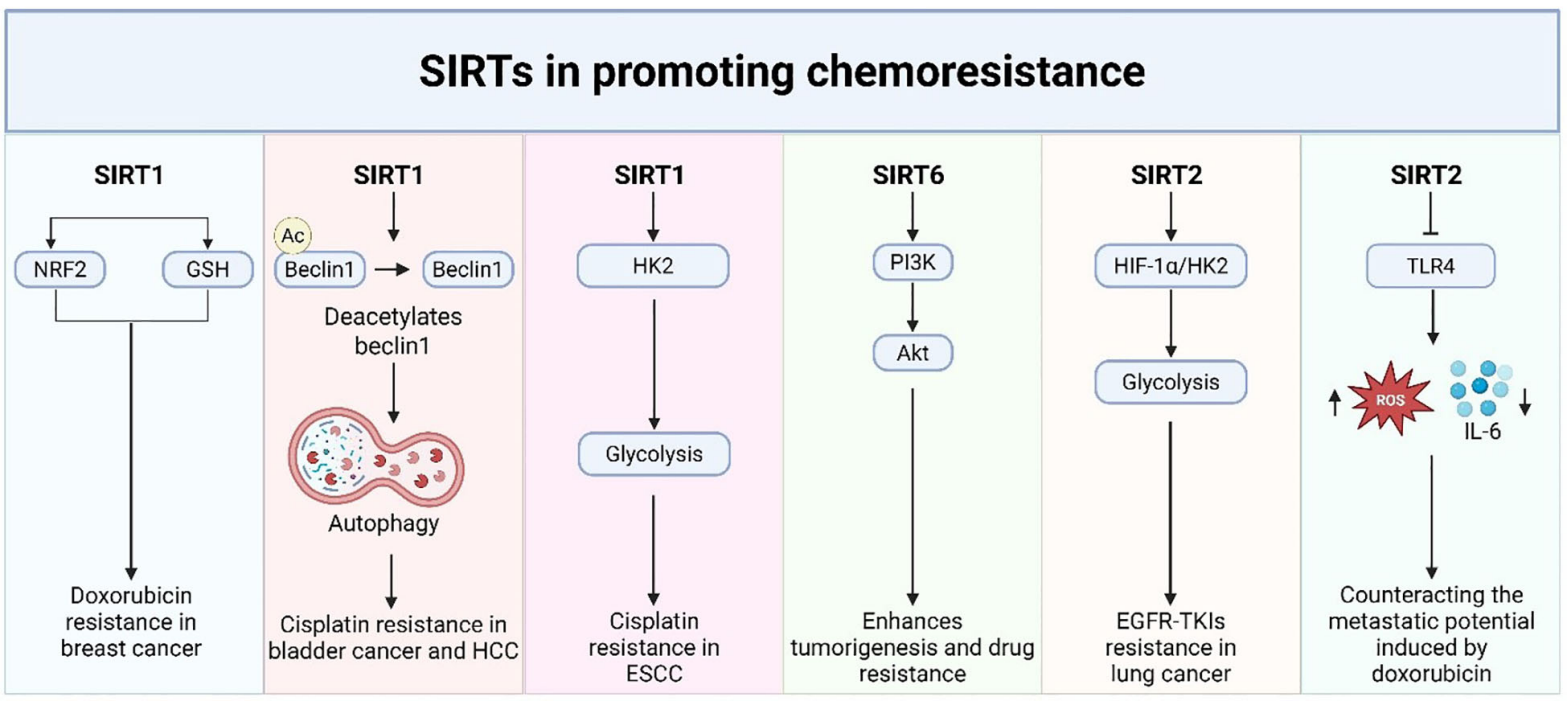
Mechanistic roles of SIRT1, SIRT2, and SIRT6 in promoting chemoresistance across various cancer types through pathways including autophagy, redox balance, EMT, metabolic reprogramming, and inflammation modulation.

Recent investigations have progressively identified members of the sirtuin family, notably SIRT1 and SIRT2, as key regulators of multiple adaptive strategies used by tumor cells to avoid chemotherapy-induced lethal effects. These multifaceted processes encompass induction of autophagy, redox balance, EMT, metabolic reprogramming, and TME modulation (**Figure 6**, **Table 6**). In NSCLC, hypoxia suppresses SIRT1 and AMPK, disrupting mitochondrial apoptosis and inducing resistance to cisplatin and doxorubicin. Pharmacologic activation of SIRT1 with SRT1720 restores chemosensitivity, highlighting a hypoxia-responsive SIRT1–AMPK axis (113). Both SIRT1 and AMPK were decreased under hypoxia, causing drug resistance to cisplatin and doxorubicin. In addition to discovering a hypoxia-sensitive SIRT1–AMPK–mitochondrial apoptosis axis, their research provided proof that SIRT1 was restored with SRT1720 to restore drug sensitivity, a discovery of immediate translational importance (113). Concurrently, Yang et al. investigated the role of SIRT6 in diffuse large B-cell lymphoma (DLBCL) and confirmed that SIRT6 enhances tumorigenesis and drug resistance by activating the PI3K/Akt pathway (213). Knockdown or small-molecule inhibition by OSS_128167 enhanced chemotherapy-induced apoptosis, inhibited proliferation, and inhibited PI3K signaling *in vitro* and *in vivo* (213). These data most conclusively identify SIRT6 as a potential therapeutic target for hematologic cancers. Building on SIRT6’s role in solid tumors, You and colleagues depicted how the deacetylase is involved in erlotinib resistance to NSCLC by metabolic reprogramming (211). SIRT6 promotes glycolysis through the HIF-1α/HK2 pathway, and SIRT6 inhibition reverses drug resistance to EGFR-TKIs (211). Their combined *in vitro* and *in vivo* studies highlight the therapeutic value of inhibiting SIRT6-mediated metabolic adaptations to overcome drug resistance in lung cancer (211).

**Table 6:** Dual roles of sirtuins in mediating chemoresistance and protecting normal tissues against chemotherapy-induced toxicities.

Cancer type	Study type	Mechanisms involved	Conclusion	Ref.
Breast Cancer	*In vitro*, *in vivo*, and SCID mice	SIRT1-mediated NRF2 nuclear translocation, GSH-mediated redox homeostasis, angiogenesis	SIRT1 promotes Dox resistance, tumor growth, angiogenesis, and metastasis via redox dysregulation; inhibiting SIRT1 or GSH reverses these effects.	(200)
Breast Cancer	In silico, *in vitro*, *in vivo* (zebrafish xenograft)	SIRT1 inhibition, apoptosis and cell cycle arrest, SIRT1-AKT-S1PR1-GNAI1/GNAO1-Tubulin axis	Selisistat enhances paclitaxel efficacy through synergistic anti-tumor effects via SIRT1 inhibition.	(201)
Breast Cancer	*In vitro*	SIRT2 inhibition, NF-κB/RelA signaling	Doxorubicin induces apoptosis by inhibiting SIRT2 and NF-κB p65 phosphorylation.	(202)
Breast Cancer (Drug resistance)	MCF-7 and patient samples	SIRT6, FOXO acetylation and DNA repair	SIRT6 confers resistance to paclitaxel and epirubicin via FOXO modulation and DNA repair enhancement.	(203)
TNBC	*In vitro* and database analysis	Targeting TLR4/SIRT2 axis, IL6 secretion from tumor-associated macrophages, oxidative stress, cellular uptake via LPS-PEC nanomicelles	Co-targeting TLR4/SIRT2 with DOX@LPS-PEC and Sirtinol reduces TNBC metastatic potential by enhancing cytotoxicity, reducing migration, and hampering IL6 secretion.	(204)
HCC	*In vitro* and sorafenib-resistant cell models	SIRT1 regulation of autophagy and NF-ĸβ pathways, deacetylation of FOXO3, Beclin1, ATGs, LC3, and p65 subunit	SIRT1 promotes autophagy and NF-ĸβ activation in sorafenib-resistant HCC, contributing to drug resistance.	(205)
Oesophageal Cancer	*In vitro*, *in vivo*, and xenograft mice	SIRT1 regulation of autophagy and EMT, tumor growth	SIRT1 upregulation in drug-resistant cells promotes autophagy and EMT; SIRT1 inhibition suppresses autophagy, EMT, and tumor growth.	(26)
ESCC	Patient samples and *in vitro*	SIRT1 expression, apoptosis, cell viability	High SIRT1 expression is linked to CRT resistance; SIRT1 knockdown enhances cisplatin and radiation sensitivity.	(206)
ESCC	*In vitro*, *in vivo*, and xenograft mice	SIRT1 regulation of glycolysis pathway, HK2 expression	SIRT1 enhances cisplatin resistance via glycolysis; SIRT1 knockdown increases chemotherapeutic sensitivity.	(207)
Ovarian Carcinoma	*In vitro*	Cytoplasmic SIRT1, polyploid giant cancer cell (PGCC) formation, senescence escape, CDK1/CDK2 signaling	Cytoplasmic SIRT1 promotes PGCC formation and survival, enhancing paclitaxel resistance.	(208)
Ovarian Cancer	*In vitro*, *in vivo*, and clinical data analysis	ACSS2 inhibition, SIRT1-mediated deacetylation of ATG5/ATG2B, glycolysis suppression, autophagy activation	ACSS2 inhibition suppresses glycolysis, activates SIRT1/ATG5/ATG2B axis, and induces autophagy, reducing malignancy and chemoresistance; paeonol targets ACSS2 to mediate this effect.	(209)
Ovarian Cancer (Cisplatin Resistance)	*In vitro* and clinical correlation	SIRT5-mediated ROS regulation, Nrf2/HO-1 signaling	SIRT5 promotes cisplatin resistance by suppressing ROS-mediated DNA damage via Nrf2/HO-1 pathway.	(27)
Bladder Cancer	*In vitro*	SIRT1-mediated Beclin1 deacetylation, autophagy activation	SIRT1 promotes cisplatin resistance via Beclin1 deacetylation-mediated autophagy; SIRT1 silencing reduces resistance.	(210)
NSCLC	Experimental (*in vitro* and *in vivo*, NSCLC-bearing mice)	SIRT6-mediated glycolysis via HIF-1α/HK2 axis, apoptosis	SIRT6 promotes erlotinib resistance through glycolysis; SIRT6 inhibition enhances erlotinib sensitivity and apoptosis.	(211)
NSCLC	*In vitro*, *in vivo*, and xenograft mice	SIRT1-AMPK pathway, hypoxia-induced chemoresistance, mitochondrial apoptosis	Hypoxia inactivates SIRT1-AMPK, leading to cisplatin/doxorubicin resistance; SIRT1 activation enhances CRT efficacy.	(113)
Lung Cancer (Cisplatin resistance)	*In vitro* and *in vivo*	SIRT3/FOXO3/CDT1 axis	SIRT3 enhances cisplatin sensitivity by regulating FOXO3-mediated CDT1 expression and inhibiting proliferation.	(212)
DLBCL	*In vitro*, *in vivo*, and xenograft mice	SIRT6-mediated PI3K/Akt signaling, apoptosis, cell cycle arrest	SIRT6 overexpression promotes tumorigenesis and drug resistance; SIRT6 inhibition enhances chemotherapy sensitivity.	(213)
CRC	*In vitro*	Cisplatin inhibition of SIRT3-mediated deacetylation of MTHFD2, redox imbalance	Cisplatin-induced SIRT3 suppression leads to MTHFD2 acetylation, disturbing redox balance and suggesting MTHFD2 as a therapeutic target.	(214)
Atypical Teratoid/Rhabdoid Tumors	Patient-derived xenografts and *in vitro*	SIRT1 repression of p53 and NF-κB, GLI2 translocation in SHH subtype	Gemcitabine promotes cell death in ATRTs via SIRT1 degradation and p53/NF-κB activation.	(215)
Pancreatic Cancer	RNA-seq, ATAC-seq, *in vivo* and xenografts	SIRT7/GLUT3/H3K122 succinylation axis	SIRT7 knockdown sensitizes PC cells to gemcitabine via GLUT3 upregulation; fasting enhances effect.	(216)
Upper Tract Urothelial Carcinoma (UTUC)	Patient tumor samples	SIRT7 expression as a prognostic marker	High SIRT7 expression correlates with poor prognosis in UTUC patients receiving platinum-based chemotherapy.	(217)
Head and Neck Squamous Cell Carcinoma	*In vitro* and 3D organotypic models	SIRT7 knockdown, EMT, cell cycle regulation	SIRT7 depletion reduces cancer cell proliferation and enhances 5-FU-induced apoptosis.	(218)
Cisplatin-induced Ototoxicity	*In vitro* and *in vivo*	SIRT3 regulation of PFKFB3-dependent glycolysis, ROS mitigation	SIRT3 protects against cisplatin-induced cochlear cell apoptosis via glycolysis regulation and ROS suppression.	(219)
Peripheral Neuropathy (Paclitaxel-induced)	*In vivo* and *in vitro*	SIRT3 activation, MnSOD2 and Nrf2 pathway	Nicotinamide riboside activates SIRT3, protecting neurons from PTX-induced damage and improving anticancer effect.	(220)
Peripheral Neuropathy (Cisplatin-induced)	*In vivo* and *in vitro*	SIRT2-mediated TC-NER repair of DNA damage	SIRT2 protects neurons by enhancing nucleotide excision repair without affecting tumor response.	(221)
Chemotherapy-Induced Cognitive Impairment	Computational and *in vitro* (SH-SY5Y cells)	NAMPT/SIRT1 pathway modulation	Quercetin and derivatives protect neurons by enhancing NAMPT/SIRT1 signaling and reducing chemobrain effects.	(222)
Kidney Damage (Cisplatin-induced)	*In vitro* and *in vivo*	SIRT3/PGC-1α pathway, mitophagy via PINK-1 and Parkin-2	Linagliptin activates SIRT3/PGC-1α and mitophagy pathways to protect kidneys from cisplatin toxicity.	(223)
Kidney Injury (Cisplatin-induced AKI)	*In vitro* and *in vivo*	SIRT3 activation by silybin, mitochondrial protection	Silybin activates SIRT3 to protect against AKI by improving mitochondrial function and reducing apoptosis.	(224)
Acute Kidney Injury (Cisplatin-induced)	*In vitro* and *in vivo*	SIRT6 inhibition of ERK1/2 via H3K9 deacetylation	SIRT6 protects against cisplatin-induced AKI by suppressing ERK1/2 signaling; a potential renal protector.	(225)
Acute Kidney Injury (Cisplatin-induced)	*In vitro* and *in vivo*	SIRT6/BAP1/xCT axis, ferroptosis inhibition	SIRT6 suppresses ferroptosis and alleviates AKI via chromatin remodeling at BAP1 promoter; potential therapeutic target.	(28)
Acute Kidney Injury (Cisplatin-induced)	*In vivo*	SIRT7/NF-κB/TNF-α signaling	SIRT7 knockout reduces TNF-α-mediated inflammation and protects against cisplatin-induced AKI.	(226)
Chondrocyte Damage (Cisplatin-induced)	*In vitro*	SIRT1/PGC-1α/Nrf2/HO-1 axis suppression	Cisplatin inhibits SIRT1-Nrf2 signaling, increasing oxidative stress and apoptosis in chondrocytes.	(227)
Nephrotoxicity (Doxorubicin-induced)	*In vitro* and *in vivo*	SIRT1/Nrf2/NF-κB/TNF-α pathways	Incretin-based therapies (ALO, SEM) protect kidneys via SIRT1-mediated antioxidant and anti-inflammatory mechanisms.	(228)
Nephrotoxicity (Cisplatin-induced)	*In vitro*	SIRT1 suppression, NF-κB p65 acetylation	SIRT1 overexpression reduces NF-κB acetylation and protects renal cells from cisplatin-induced injury.	(229)
Nephrotoxicity (Cisplatin-induced)	*In vivo*	SIRT1/p53/FOXO3a/Nrf2/NF-κB pathway modulation	Theaflavin protects against cisplatin-induced nephrotoxicity by regulating SIRT1-related antioxidant and anti-inflammatory signaling.	(230)
Cardiotoxicity (Doxorubicin-induced)	Experimental (mechanistic study)	SIRT6-mediated Nrf2/FUNDC1 signaling, mitochondrial protection	SIRT6 activation protects against doxorubicin-induced cardiomyopathy by enhancing mitochondrial biogenesis and reducing oxidative stress.	(231)
Cardiotoxicity/Multiple Cancer Types	*In vitro*, *in vivo*, and patient data	SIRT6 activation, metabolic remodeling, mitophagy, Sgk1 inhibition	SIRT6 overexpression alleviates doxorubicin-induced cardiotoxicity and enhances anticancer efficacy via metabolic remodeling.	(232)
Cardiotoxicity (Doxorubicin-induced)	*In vitro* and *in vivo*	SIRT4-mediated inhibition of autophagy via Akt/mTOR pathway	SIRT4 overexpression protects heart by inhibiting excessive autophagy and improving cardiac function.	(233)
Cardiotoxicity (Doxorubicin-induced)	*In vitro*, *in vivo*, and knockout models	SIRT1/SESN2 interaction and MDM2-mediated ubiquitination suppression	SIRT1 stabilizes SESN2, reducing oxidative damage and apoptosis in doxorubicin-treated hearts.	(234)
Cardiotoxicity (Doxorubicin-induced)	*In vitro* and *in vivo*	SIRT3 inhibition of NLRP3 via mTOR/ULK1 pathway and autophagy regulation	SIRT3 attenuates cardiotoxicity by inhibiting inflammasome activation through autophagy modulation.	(235)
Cardiotoxicity (5-FU-induced)	*In vivo*	SIRT1/Nrf2/HO-1 pathway	Taxifolin protects against 5-FU-induced cardiotoxicity by modulating oxidative stress and apoptosis.	(236)
Cardiotoxicity (Doxorubicin-induced)	*In vitro* and *in vivo*	Rnd3/Rock1/Drp1/mitochondrial fission, PANoptosis	Rnd3 stabilizes mitochondrial dynamics and prevents DOX-induced PANoptosis, reducing cardiotoxicity.	(237)
Cardiotoxicity (Doxorubicin-induced)	*In vitro* and *in vivo*	HRD1-mediated Nrf2 degradation inhibition	Tirzepatide reduces DOX-induced cardiotoxicity by stabilizing Nrf2 and reducing oxidative stress.	(238)
Cardiotoxicity (Doxorubicin-induced)	*In vitro* and *in vivo*	SIRT7 activation, cuproptosis inhibition	Aprocitentan protects heart by activating SIRT7 and reducing cuproptosis and oxidative damage.	(239)
Cardiotoxicity (CHK1 + Gemcitabine-induced)	*In vitro* and *in vivo*	SIRT3-dependent mitochondrial redox regulation	SIRT3 maintains mitochondrial function and reduces pyroptosis in gemcitabine-induced cardiotoxicity.	(240)
Cardiomyopathy (Doxorubicin-induced)	*In vivo*, and gene expression study	SIRT1/Nrf2/HO-1 and ABCB4 pathways	Edaravone improves cardiac function and survival by activating Nrf2 and SIRT1-mediated antioxidant pathways.	(241)
Hepatotoxicity (Doxorubicin-induced)	*In vitro* and *in vivo*	SIRT1/Nrf2/HO-1 upregulation, NF-κB suppression	Vildagliptin protects liver by activating antioxidant pathways and reducing inflammation and apoptosis.	(242)
Hepatotoxicity (Doxorubicin-induced)	*In vitro* and *in vivo*	SIRT1/AMPK upregulation, NF-κB/NLRP3 inflammasome suppression	Febuxostat protects liver via antioxidative and anti-inflammatory SIRT1/AMPK pathway activation.	(243)
Cardiac Mitochondrial Dysfunction	*In vitro*	SIRT3-AMPKα-PGC-1α axis, mitochondrial biogenesis and dynamics	SIRT3 promotes mitochondrial health and biogenesis, enhancing cardiac energy metabolism and reducing oxidative stress.	(244)
Intestinal Mucositis (5-FU-induced)	*In vivo*	AMPK/SIRT1, PI3K/AKT, TLR4/NF-κB/MAPK axis	Chlorogenic acid alleviates mucositis by activating SIRT1 and reducing inflammation and oxidative stress.	(245)
Endothelial Injury (5-FU-induced)	*In vitro*	SIRT1 activation, oxidative stress and senescence modulation	Echinacoside mitigates 5-FU-induced endothelial injury via SIRT1 pathway activation.	(246)

Sun et al. reported that SIRT1 increases cisplatin resistance in bladder cancer by deacetylating Beclin1, which induces greater autophagy (210). Sensitivity was restored by either inhibiting SIRT1 or autophagy, underscoring the importance of the interaction between Beclin1 and SIRT1 for cell survival during chemotherapy (210). In HCC, SIRT1 also mediated autophagy activation and NF-κB suppression in sorafenib-resistant cells (205). Its repression not only destabilizes autophagy by FOXO3 and ATG proteins but also reinstates NF-κB transcriptional activity, revealing a double regulatory loop (205). Redox homeostasis is another critical process of resistance. Sahoo et al. have demonstrated that in breast cancer, SIRT1 enhances NRF2 translocation and GSH generation, thereby reinforcing doxorubicin resistance, tumor angiogenesis, and metastasis (200). Inhibition of SIRT1 or inhibition of GSH formation reversed them, once more establishing the redox-protective function of SIRT1 (200).

SIRT1’s impact extends to ovarian cancer, wherein Xu et al. indicated that its cytoplasmic localization promotes the formation and survival of polyploid giant cancer cells (PGCCs) after paclitaxel treatment (208). PGCCs are less senescent and are responsible for drug tolerance and recurrence (208). In esophageal cancer, Zhang et al. observed that SIRT1 promotes autophagy and EMT, thereby enhancing the migratory capability of resistant cells (26). Knockdown of SIRT1 disrupted both signaling pathways and compromised cell motility (26). Another study demonstrated that the overexpression of SIRT1 in ESCC tissues is associated with CRT resistance, and that SIRT1 knockdown sensitizes the cells to radiation and cisplatin (206). Yang et al. confirmed the connection between SIRT1 and metabolic reprogramming, showing that SIRT1 confers resistance in ESCC by upregulating HK2, a key enzyme in glycolysis, thereby increasing tolerance to chemotherapy (207). SIRT1 silencing impaired glycolytic flux and restored sensitivity to cisplatin (207). While less well characterized, SIRT2 is important too. Mahmoud et al. demonstrated that silencing the TLR4/SIRT2 pathway, achieved in TNBC using Sirtinol-conjugated nanomicelles, resulted in increased oxidative stress, inhibition of IL-6 secretion by tumor-associated macrophages, and blockade of cancer cell migration, effectively counteracting the metastatic potential induced by doxorubicin (204).

Collectively, the growing evidence suggests that sirtuin-mediated chemoresistance is achieved through a finite number of conserved signaling pathways that integrate metabolic, redox, and survival pathways across various cancer types. Among these, the activation of the PI3K/Akt pathway by SIRT6 has been identified as a central pro-survival mechanism that enhances tumor growth and drug resistance in diffuse large B-cell lymphoma (213). Another important regulatory pathway is the NF-κB pathway, in which the deacetylation and repression of NF-κB signaling by SIRT1 promote adaptive redox balance and anti-apoptotic responses in sorafenib-resistant HCC (205). Moreover, the AMPK/mTOR axis provides a critical interface between metabolism and autophagy. SIRT1 functions with AMPK to regulate mitochondrial apoptosis and drug sensitivity in NSCLC (113), while SIRT3 modulates the mTOR/ULK1 pathway to maintain autophagic flux and reduce chemotherapy-induced stress (235). Meanwhile, the HIF-1α/ROS regulatory axis couples the SIRT3- and SIRT6-driven metabolic reprogramming to oxidative adaptation and glycolytic flexibility, enabling tumor cells to maintain mitochondrial function and survive cytotoxic insults (211, 214). Taken together, these interconnecting cascades demonstrate that different SIRT isoforms converge on a common signaling platform, including PI3K/Akt, NF-κB, AMPK/mTOR, and HIF-1α/ROS, which collectively promote chemotherapy resistance, as comprehensively summarized in **Table 6**.

### 7.2 Mechanisms enhancing chemosensitivity

**Figure 7:**
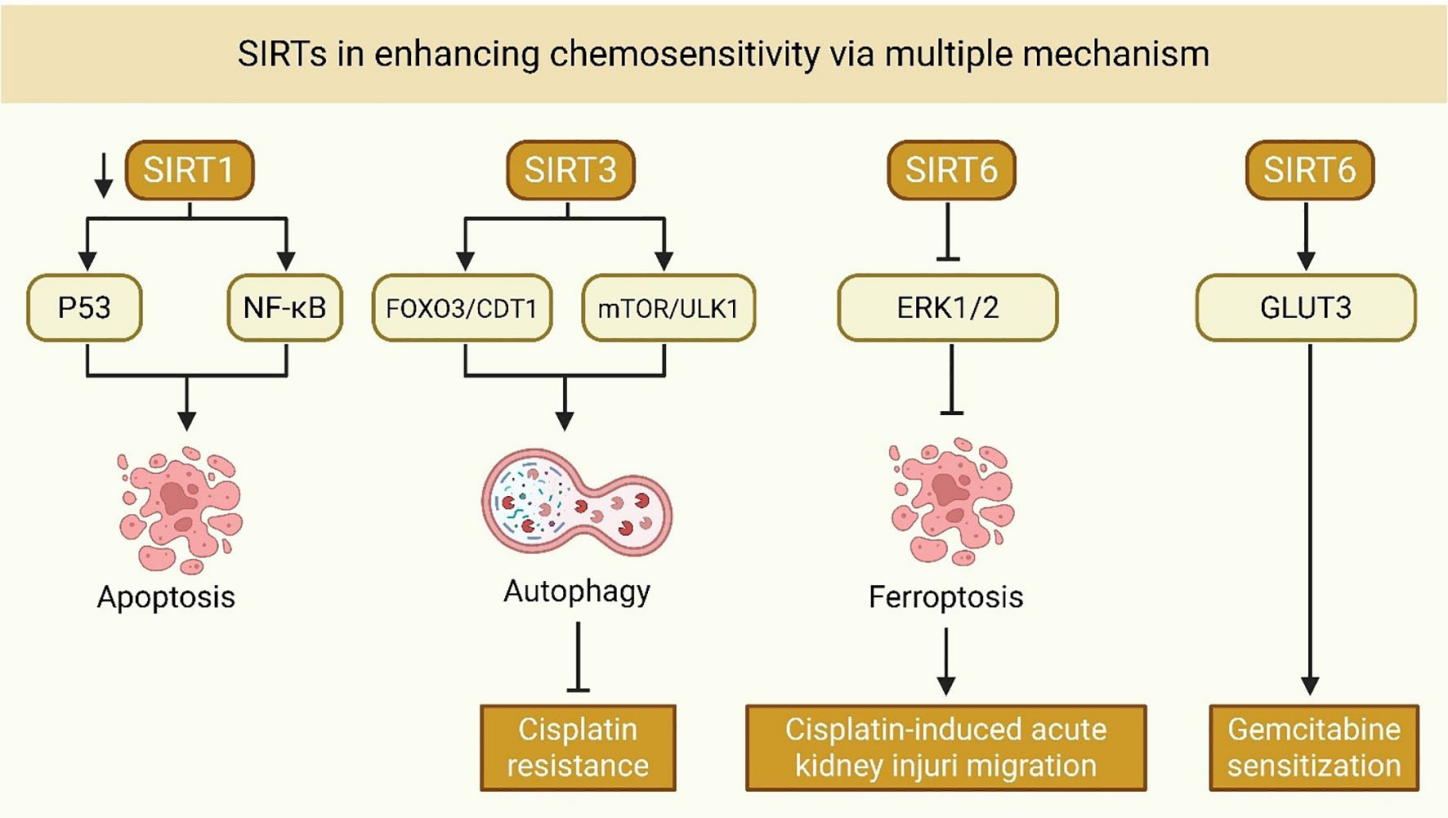
Sirtuins enhance chemosensitivity through diverse mechanisms including apoptosis induction (SIRT1), autophagy restoration and cardioprotection (SIRT3), kidney protection via ERK1/2 and ferroptosis inhibition (SIRT6), and increased drug uptake through GLUT3 upregulation (SIRT7), highlighting their context-dependent roles in optimizing chemotherapy outcomes.

Enhancing chemosensitivity is still a major problem for cancer treatment, particularly in the context of drug resistance and normal tissue collateral damage. Several recent studies have identified various molecular mechanisms that sensitize tumors while sparing normal tissues, thereby enhancing the overall therapeutic index of chemotherapy (**Figure 7**, **Table 6**). Li et al. showed that overexpression of SIRT6 mitigated cisplatin-induced acute kidney injury by repressing ERK1/2 signaling through histone deacetylation (225). Beyond organ protection, this indicates that SIRT6 may help maintain the systemic viability required for sustained chemotherapy regimens (28). Yang et al. found that SIRT6 suppresses ferroptosis in renal tissue through the BAP1/xCT axis, again making it a cellular resistance factor to chemotoxic stress (28). These two regulatory effects mean that SIRT6 indirectly sensitizes cells to chemotherapy by keeping collateral off-target organ damage in check.

In cancer, SIRT3 was first postulated as a sensitizing agent in lung cancer. Cao et al. demonstrated that the overexpression of SIRT3 inhibits cisplatin resistance by activating the FOXO3/CDT1 pathway, thereby enhancing cell proliferation and invasion (212). A similar sensitizing effect of SIRT3 in cardiomyocytes was also presented by Sun et al., who reduced doxorubicin-induced cardiotoxicity by inhibiting the NLRP3 inflammasome and restoring autophagy by the mTOR/ULK1 pathway (235). This dual function—tumor inhibition and protection of host tissue—highlights the therapeutic value of SIRT3 activation in chemotherapy.

SIRTs can also function as inhibitory targets to enhance the efficacy of chemotherapeutic agents. Chen et al. revealed that SIRT7 knockdown increased the sensitivity of pancreatic cancer cells to gemcitabine by up-regulating GLUT3 (216). They demonstrated that SIRT7 directly represses GLUT3 transcription through histone desuccinylation, and in the case of SIRT7 deletion, significantly increases drug entry and efficacy, even under fasting conditions (216). Moreover, Metselaar et al. demonstrated that gemcitabine enhances SIRT1 degradation in atypical teratoid rhabdoid tumors (ATRT), which in turn reactivates p53 and NF-κB signaling, promoting apoptosis, particularly in SHH-subtype tumors (215). Such studies align with the hypothesis that some sirtuin inhibition can disrupt tumor resistance networks. Wawruszak et al. pursued this further by screening selisistat, a selective SIRT1 inhibitor, with paclitaxel in models of breast cancer (201). Combination therapy resulted in additive anti-tumor activity, including enhanced apoptosis and inhibition of growth, which was greater than that of either drug given alone (201). Notably, in silico analysis demonstrated that SIRT1 was linked to tubulin-associated pathways via AKT and S1PR1 signaling, thereby providing a mechanism for synergy (201).

Selective activation or inhibition of specific SIRT isoforms, depending on the tissue context, can enhance chemosensitivity through diverse mechanisms, including the control of ferroptosis, modulation of autophagy, and metabolic reprogramming. Interestingly, this evidence as a whole suggests that finely regulated SIRTs will maximize anti-tumor effectiveness while simultaneously sparing essential organs, thereby extending the therapeutic window. Follow-up studies will be directed towards the design of sirtuin isoform-specific modulators and the assessment of their combinatory value with known chemotherapy drugs in tissue-specific ways.

### 7.3 Metabolic reprogramming

**Figure 8:**
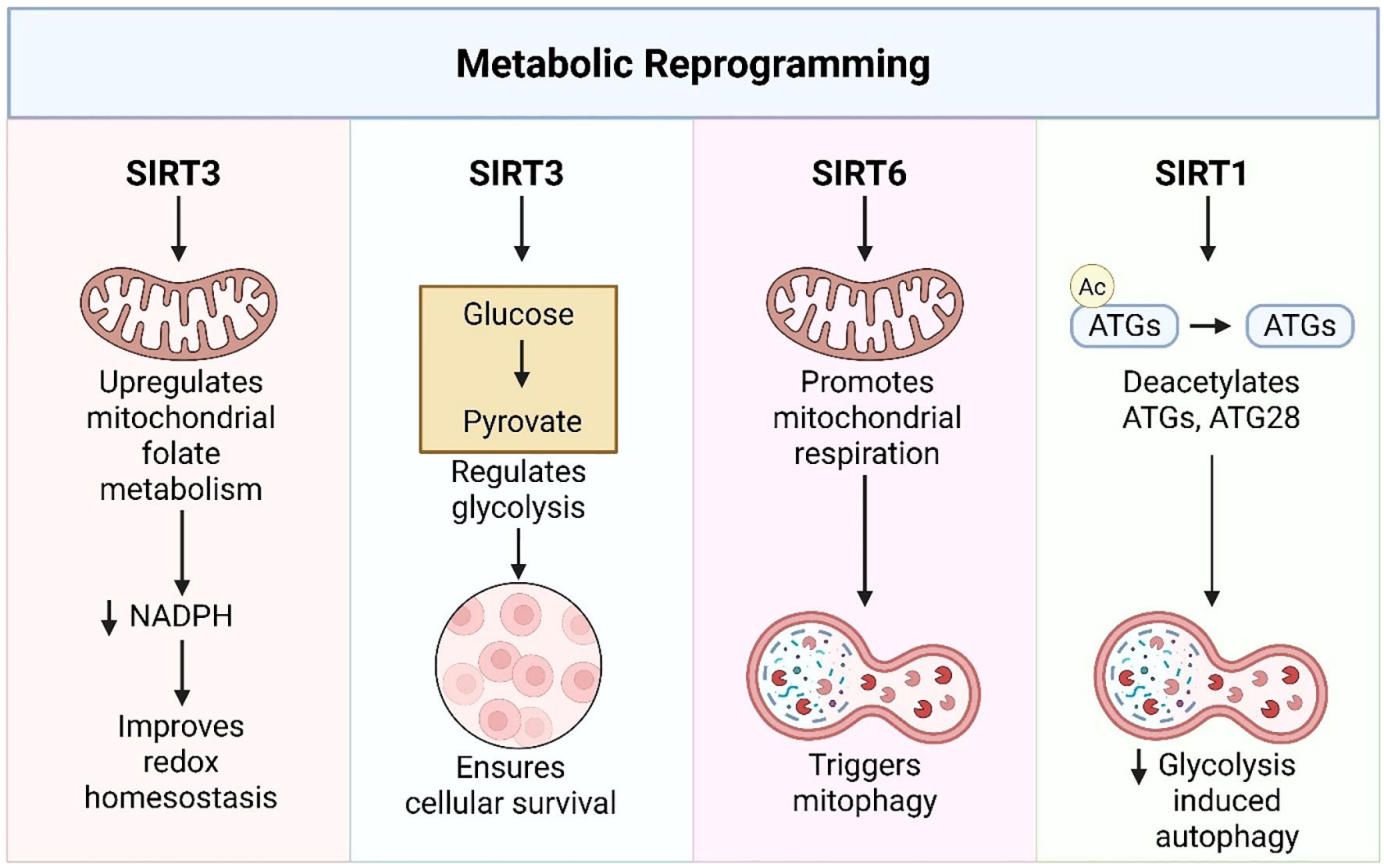
Sirtuins orchestrate metabolic reprogramming under chemotherapeutic stress. SIRT3 enhances mitochondrial folate metabolism and glycolysis to maintain redox balance and cell survival. SIRT6 promotes mitochondrial respiration and mitophagy, while SIRT1 induces autophagy by deacetylating key glycolytic regulators, collectively supporting adaptive metabolic responses.

Metabolic reprogramming is a hallmark of cancer that sustains the high biosynthetic and energetic requirements for proliferation-driven growth of tumor cells. In addition to the classical Warburg effect, accumulating evidence suggests that cancer cells undergo adaptive alterations in mitochondrial metabolism, redox state, and nutrient utilization to endure therapy-induced stress and survive. Sirtuins, particularly SIRT3 and SIRT6, are key regulators of adaptive responses and direct chemoresistance through metabolic control (**Figure 8**, **Table 6**). Wan et al. also demonstrated that cisplatin suppresses SIRT3 in CRC cells, resulting in hyperacetylation and inactivation of the key enzyme MTHFD2, which is involved in mitochondrial folate metabolism (214). Consequently, NADPH generation is disrupted, along with redox homeostasis, making the cells vulnerable to oxidative stress (214). Bugga et al. identified that SIRT3 controls mitochondrial biogenesis and function via AMPKα–PGC-1α signaling, driving ATP production and antioxidant defense mechanisms (244). Likewise, Ewees et al. reported that in a cisplatin-induced rat model of nephrotoxicity, the anti-diabetic medication linagliptin acts as a mitophagy inducer, triggering the SIRT3/PGC-1α pathway (223). This is distinguished by increased expression of PINK1 and Parkin, the two regulators of mitochondrial quality control. Linagliptin also enhanced FOXO3 activity and suppressed inflammatory cytokines, suggesting that SIRT3 triggers a multifaceted protective mechanism under chemotherapy-induced metabolic stress (223).

Consistent with this finding, Tang et al. identified a novel role of SIRT3 in preventing cisplatin-induced ototoxicity (219). They identified that SIRT3 controls PFKFB3-dependent glycolysis in cochlear cells. SIRT3 knockdown was shown to impair glycolytic metabolism and increase ROS accumulation, whereas overexpression guaranteed glycolytic activity and cell viability (219). These findings are consistent with the hypothesis that SIRT3 controls mitochondrial and cytoplasmic metabolic networks to prevent chemotoxicity. In another related study, Sun et al. demonstrated that nicotinamide riboside induced SIRT3 activation, thereby rescuing paclitaxel-induced oxidative stress in peripheral neurons. Anticancer activity was through the MnSOD2 and Nrf2 pathway (220).

Peng et al. demonstrated that SIRT6 overexpression reduces doxorubicin-induced cardiotoxicity while increasing its antitumor efficacy (232). Mechanistically, SIRT6 reprograms energy metabolism from glycolysis towards mitochondrial respiration by inhibiting SGK1 and inducing mitochondrial biogenesis and mitophagy (232). This bimodal effect, in which SIRT6 protects normal tissues while sensitizing cancer cells, again points to its candidate status as a context-dependent metabolic regulator. Wang and colleagues further extended this observation to show that SIRT6 also activates the Nrf2/FUNDC1 pathway, increasing mitochondrial metabolism in doxorubicin-treated cardiomyocytes and thereby counteracting necrosis and apoptosis (231). On the glycolytic side, Yang and colleagues demonstrated that inhibition of ACSS2 in ovarian cancer impairs acetate metabolism and glycolysis, leading to the activation of nuclear SIRT1 (209). This resulted in the deacetylation of ATG5 and ATG2B, leading to the induction of autophagy and the suppression of chemoresistance (209). The therapeutic activity of paeonol was also reproduced along the ACSS2/SIRT1 pathway (209). These findings position SIRTs as key integrators of metabolic remodelers in tumor and non-tumor tissue. SIRT3 and SIRT6, in particular, help make chemotherapy more effective while keeping healthy tissue safe by protecting the mitochondria. Their tumor-supportive activity in certain situations, however, does require precision-based targeting. Overall, the way SIRTs regulate metabolism is a promising area for new treatments that warrant further exploration.

### 7.4 DNA repair and redox balance

**Figure 9:**
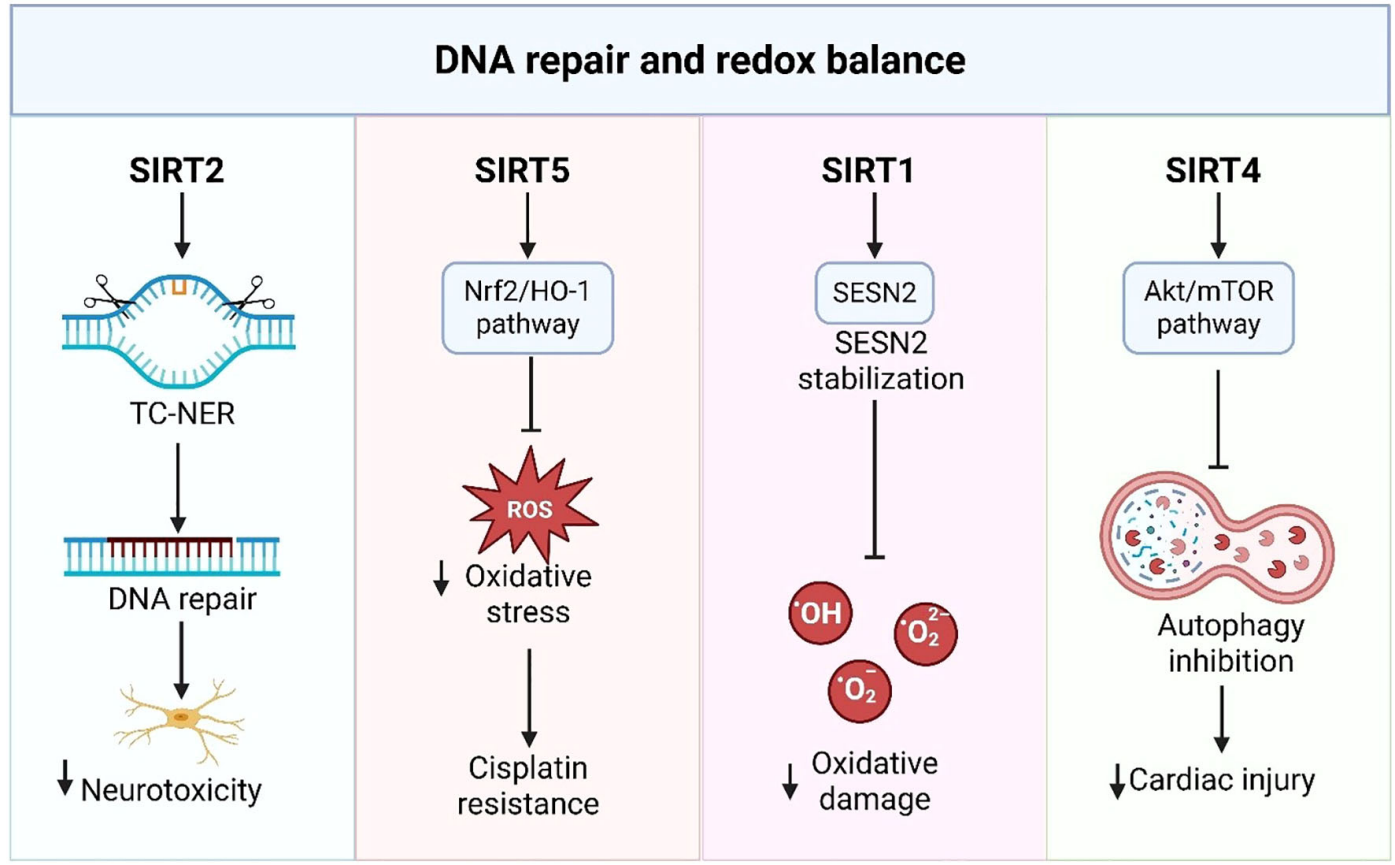
Sirtuins contribute to DNA repair and redox balance through isoform-specific roles. SIRT5 promotes cisplatin resistance via the Nrf2/HO-1 antioxidant pathway. In contrast, SIRT2 supports neuroprotection by enhancing TC-NER-mediated DNA repair. SIRT1 stabilizes SESN2 to reduce oxidative damage, while SIRT4 inhibits autophagy through the Akt/mTOR axis, mitigating cardiac injury. These findings highlight the dual roles of sirtuins in both protecting normal tissues and modulating tumor sensitivity to chemotherapy.

Genomic stability and redox homeostasis are crucial during chemotherapy, as DNA damage and oxidative stress are key factors in the cytotoxicity of agents such as cisplatin and doxorubicin. New research has focused on the function of sirtuins, NAD+-dependent deacetylases, as regulators of these events of utmost importance. In ovarian cancer, SIRT5 activates the Nrf2/HO-1 pathway to alleviate ROS accumulation and DNA damage, thereby conferring cisplatin resistance through enhanced redox buffering (27). In contrast, Zhang et al. determined that SIRT2 plays a therapeutic role in non-malignant tissue, enhancing TC-NER in peripheral neurons and repressing cisplatin-induced neurotoxicity (**Figure 9**, **Table 6**) (221). The results suggest the two-edged nature of sirtuins: whereas certain isoforms (e.g., SIRT5) defend malignant cells, thereby undermining therapy, others, such as SIRT2, are involved in tissue-specific protection without promoting tumor survival.

The connection between redox balance and DNA repair is established in research on doxorubicin-induced toxicities. Wang et al. demonstrated that SIRT1 in the heart stabilizes SESN2 and suppresses oxidative damage in cardiomyocytes (234), and that He et al. rendered SIRT4 a negative regulator of autophagy by targeting the Akt/mTOR pathway (233), and reducing cardiac injury. Both studies demonstrate that modulation of oxidative stress pathways also reinforces more general stress response mechanisms, ultimately maintaining DNA integrity.

The position of the Nrf2 pathway as a downstream target for sirtuin action is a recurring theme. Botros et al. (228) and Yoshikawa et al. (241) demonstrated that activating SIRT1 reactivated Nrf2 signaling, thereby reducing oxidative stress and protecting kidney and heart functions in tissues treated with doxorubicin. Similarly, the protective effects of vildagliptin and febuxostat on the liver were associated with increased SIRT1 levels and its related antioxidant proteins, HO-1 and NQO-1, a common process observed in various tissues (212, 225), suggesting a conserved mechanism across different tissues. Conversely, Hsieh et al. highlighted the role of downregulation of SIRT1 in cisplatin-exposed chondrocytes (227). Mitochondrial damage, enhanced oxidative stress, and activation of pro-inflammatory pathways, leading to the disassembly of the tissue matrix, resulted from the disruption of the SIRT1/PGC-1α/Nrf2 pathway (227). This result not only aligns with SIRT1’s protective function in intact tissues but also presents support for the precise balance between redox regulation and tissue homeostasis. In summary, these studies provide a unifying mechanism of regulation whereby SIRTs promote redox homeostasis and DNA repair through Nrf2 activation, maintain mitochondrial integrity, and inhibit apoptotic signaling. Yet, the two-faced nature of SIRTs, as both guardians of normal cells and potential drivers of cancer resistance, makes them difficult to target therapeutically. An optimized strategy is called for: the selective activation of protective SIRTs in regular tissue and the blocking of tumor-stimulatory ones, such as SIRT5, would make one more sensitive to chemotherapy. Future research must focus on the development of isoform-selective modulators that respect the tissue environment and the chemotherapeutic regimen.

Although SIRT-mediated regulation of oxidative stress has been shown consistently in both tumor progression and therapy resistance, these effects are highly context-dependent and sometimes paradoxical. For instance, SIRT1 and SIRT3 may preserve redox homeostasis and protect non-malignant cells from oxidative damage, yet the same antioxidant function may also sustain tumor survival under metabolic or chemotherapeutic stress. Conversely, SIRT5-driven activation of the Nrf2/HO-1 axis enhances DNA repair and antioxidant defense in cancer cells, hence conferring drug resistance. Inhibition of SIRT5, on the other hand, sensitizes tumors to ROS-mediated apoptosis. These opposing outcomes underline the dualistic nature of SIRT signaling in oxidative stress: it is protective in normal physiology but can be oncogenic under malignant conditions. The translation of SIRT modulators into clinical oncology requires a more detailed understanding of this redox duality.

### 7.5 Context-dependent functions and therapeutic implications

**Figure 10:**
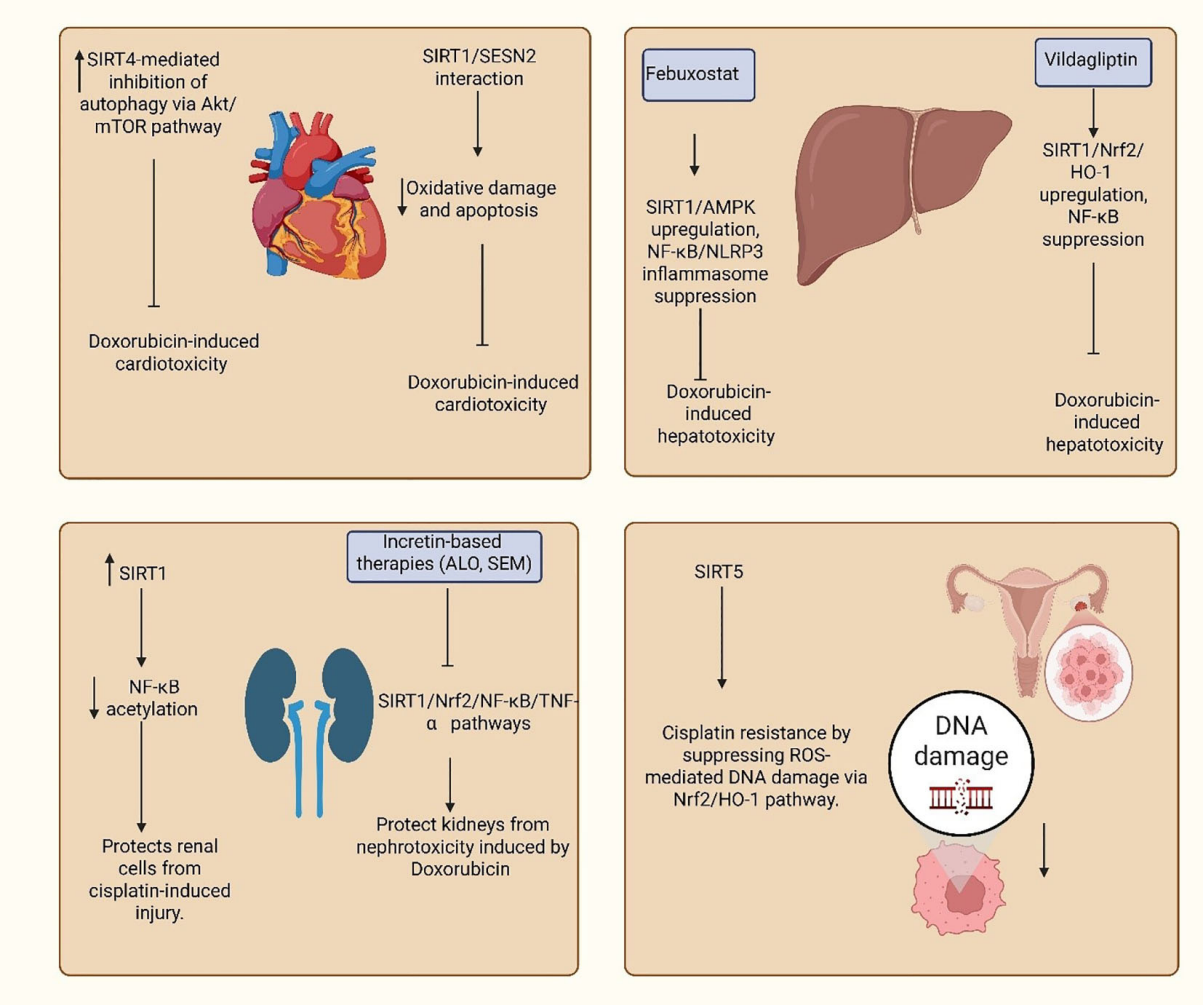
Sirtuins confer organ-specific protection against chemotherapy-induced toxicity through distinct molecular pathways. SIRT1, SIRT4, and SIRT5 mitigate chemotherapy-associated organ damage via diverse mechanisms. In the heart, SIRT4 suppresses autophagy via Akt/mTOR signaling, while the SIRT1/SESN2 axis reduces oxidative damage and apoptosis, collectively attenuating doxorubicin-induced cardiotoxicity. In the liver, febuxostat enhances SIRT1/AMPK activity and represses NF-κB/NLRP3 inflammasome signaling, whereas vildagliptin promotes SIRT1/Nrf2/HO-1 signaling and suppresses NF-κB, reducing doxorubicin-induced hepatotoxicity. In the kidney, SIRT1 deacetylates NF-κB, and incretin-based therapies (ALO, SEM) activate SIRT1/Nrf2/NF-κB/TNF-α pathways, affording protection against cisplatin- and doxorubicin-induced nephrotoxicity. In ovarian cells, SIRT5 limits cisplatin-induced cytotoxicity by suppressing ROS-mediated DNA damage through the Nrf2/HO-1 pathway.

The sirtuin family, specifically SIRT1, SIRT2, SIRT3, and SIRT7, exhibits context-dependent functions in modulating both the toxic and therapeutic effects of chemotherapeutic drugs. SIRT1 is often suggested to be a cytoprotective molecule in non-cancerous tissues exposed to chemotherapeutic injury (**Figure 10**, **Table 6**). Alanazi et al. demonstrated that theaflavin increased SIRT1 activity to induce resistance against cisplatin-induced nephrotoxicity by inhibiting p53, FOXO3a, and NF-κB signaling, as well as activating Nrf2 (230). Such renoprotective actions were observed by Miyasato et al. (246), who also noted that cisplatin-induced acute kidney injury in mice was resistant to SIRT7 knockout. This was primarily by NF-κB-dependent downregulation of TNF-α. Apart from this, Echinacoside, according to Li et al. (246), activated SIRT1 and inhibited 5-FU-induced endothelial senescence and injury once again through the SIRT1-AMPK-eNOS pathway for vascular protection. Taxifolin inhibited 5-FU-induced cardiotoxicity also through the SIRT1/Nrf2/HO-1 pathway (236). These observations not only validate the antioxidant and anti-inflammatory action of SIRT1 but also demonstrate its susceptibility to food or plant-based compounds, suggesting its status as a modifiable therapeutic node (236).

The neuroprotective and cardioprotective functions of SIRT1 have also been established. John et al. (244) demonstrated that quercetin and its derivatives activate NAMPT/SIRT1 signaling in neuronal cells, thereby alleviating cognitive dysfunction induced by chemotherapy. Likewise, Lin et al. (245) found that chlorogenic acid exhibited anti-inflammatory and antioxidant effects against 5-FU-induced mucositis through the SIRT1-mediated modulation of the TLR4/NF-κB/MAPK and PI3K/AKT pathways. SIRT3 was primarily linked to mitochondrial well-being. Chen et al. (240) provided strong evidence that SIRT3 maintained redox homeostasis in cardiomyocytes during CHK1 inhibitor and gemcitabine treatment. Deletion of mitochondrial SIRT3 led to mtROS accumulation, pyroptosis, and cardiac dysfunction, which were rescued by SIRT3 overexpression (240). These results suggest that inhibiting SIRT3 in mitochondria may be an effective and specific approach for preventing metabolic cardiotoxicity.

SIRT7 showed the most paradoxical picture, though. Aside from its renoprotective function upon knockout, higher expression of SIRT7 correlated with unfavorable prognosis in platinum-based chemotherapeutic patients with urothelial carcinoma (217). Halasa et al. (218) also demonstrated that SIRT7 ablation in head and neck squamous cell carcinoma inhibited proliferation, EMT, and resulted in S-phase arrest following 5-FU treatment. Notably, in a cardiac model, Aprocitentan reduced doxorubicin-induced cardiotoxicity by activating SIRT7, reducing oxidative stress, mitochondrial injury, and cuproptosis (239). Such dualism suggests that SIRT7 is oncogenic in cancer but protective in non-malignant tissue conditions, particularly under conditions of chemotherapeutic stress.

Other SIRTs were also involved. Doxorubicin was reported to inhibit SIRT2 and NF-κB p65 phosphorylation in murine breast cancer cells, inducing apoptosis (202). Less characterized but important, this implicates SIRT2 inhibition as a new anti-cancer mechanism. Metselaar et al. (215) also demonstrated that gemcitabine inhibits SIRT1 in ATRT tumors, thereby releasing p53 and NF-κB activity, and inducing apoptosis, uncovering a weakness in SIRT1-dependent tumors (215). Supplementing this molecular information, further drugs, such as tirzepatide (221) and Rnd3 (234), stimulated SIRT-independent pathways but shared common targets, including Nrf2 and mitochondrial protection, again echoing the significance of oxidative and stress response modulation in minimizing chemotherapy toxicity. SIRTs are not entirely protective or toxic; their activities are highly context-dependent in cells, tissues, and in response to chemotherapeutic insults. Although SIRT1 and SIRT3 are generally protective of normal tissue, inhibiting them in cancer cells could potentially enhance the effectiveness of chemotherapy. SIRT7’s tumor versus cardiac muscle dualism highlights the need for highly selective targeting strategies. Future therapeutic development should thus target tissue-specific modulation or dual-function compounds that can selectively discriminate between cancer and normal cell environments. Without such specificity, indiscriminate activation or inhibition of the SIRTs will result in unwanted side effects.

## 8 Challenges and future prospects

Despite the constantly increasing amount of research on SIRTs in cancer biology, numerous key challenges prevent their full therapeutic potential from being realized. First, the context-dependent duality of SIRT isoforms, which exhibit tumor-promoting activities in certain contexts and tumor-suppressive activities in others, makes it challenging to design universal modulators. The same SIRT, such as SIRT (155) or SIRT6 (151), can enhance immune evasion in one cancer type while suppressing metastatic progression in another (78, 92), underscoring the need for isoform-, tissue-, and disease-stage-specific strategies. Second, the lack of selective pharmacological tools remains a significant barrier. Currently available SIRT inhibitors or activators primarily affect multiple isoforms or lack adequate bioavailability and specificity (74, 247, 248). This off-target effect contributes to the risk of untargeted immunosuppression or toxicity, particularly when combined with immunotherapies or chemotherapeutics. Third, restricted clinical translation persists despite promising preclinical results. Despite extensive preclinical research in pivotal modulator-based targeted agents (227, 230), clinical translation has so far remained challenging. Research will continue to be needed in combination therapy and the development of novel strategies that overcome the inherent heterogeneity and therapeutic resistance of cancer. The integration of SIRT activity profiling into precision oncology platforms can offer improved therapeutic efficacy and reduced resistance. Future multi-omics strategies, combining transcriptomics, proteomics, and metabolomics, will help show the regulatory mechanisms underlying SIRTs within different cancers and immune contexts over the coming years. Notably, the development of intelligent systems, such as tumor-targeting nanoparticles with SIRT (249, 250) modulators, offers potentially transformative therapies through improved specificity and reduced systemic toxicity. Overall, unraveling SIRT biology and translating it into a successful treatment will require an interdisciplinary effort, improved model systems, and a patient-centered approach. The emphasis of research in the near future will focus on biomarker discovery, the selective modulation of SIRT biology, and integrating SIRT biology into immuno-oncology and combination therapy platforms. Future investigations should not aim to generalize the activation or inhibition of SIRTs, but rather to resolve their context-specific duality through integrative mechanistic mapping and translational modeling, steps that will ultimately determine the clinical safety and efficacy of SIRT modulators.

## 9 Conclusion

SIRTs constitute a highly context-dependent regulatory layer at the interface of tumor metabolism, epigenetics, and immune control. Their capacity to either suppress or promote tumor progression is dictated by isoform specificity, subcellular localization, metabolic state, and the immune composition of the TME. Throughout this review, we have highlighted how distinct SIRT isoforms influence cancer hallmarks such as metabolic rewiring, autophagy, immune evasion, and therapeutic resistance. Notably, SIRT1 and SIRT6 can reinforce immune suppressive networks and chemoresistance, whereas SIRT2, SIRT3, and SIRT7 can restore antitumor immunity and enhance therapy responsiveness under defined biological conditions.

Therapeutically, SIRTs occupy a strategic position for next-generation oncology. The main challenge ahead lies in developing isoform-selective modulators that achieve sufficient precision to avoid systemic metabolic and immune toxicity. Equally important is understanding how SIRT-driven pathways intersect with immune checkpoint regulation and metabolic plasticity during immunotherapy. This highlights the need for patient stratification guided by SIRT expression signatures, metabolic profiling, and TME state. A forward-looking oncology strategy will involve rational inhibition of immunosuppressive SIRT axes such as SIRT1/SIRT6/SIRT5 to reduce Treg dominance and immune exclusion, while supporting SIRT2 and SIRT4 activity to enhance CD8+ T cell metabolic fitness. Integration of multi-omics, high-resolution single-cell analyses, and adaptive therapeutic modeling will be essential for translating these mechanistic insights into clinically actionable frameworks. In summary, decoding the context-specific biology of SIRTs provides a promising foundation for precision immuno-metabolic cancer therapy. Continued interdisciplinary research may ultimately enable the development of SIRT-targeted interventions that align metabolic control with durable antitumor immune responses.
